# Toward Cumulative Cognitive Science: A Comparison of Meta-Analysis, Mega-Analysis, and Hybrid Approaches

**DOI:** 10.1162/opmi_a_00048

**Published:** 2021-11-25

**Authors:** Ezequiel Koile, Alejandrina Cristia

**Affiliations:** National Research University Higher School of Economics; Department of Linguistic and Cultural Evolution, Max Planck Institute for the Science of Human History; Laboratoire de Sciences Cognitives et Psycholinguistique, De´partement d’e´tudes cognitives, ENS, EHESS, CNRS, PSL University

**Keywords:** cumulative science, open science, meta-analyses, mega-analyses, data simulation, fixed effects, random effects

## Abstract

There is increasing interest in cumulative approaches to science, in which instead of analyzing the results of individual papers separately, we integrate information qualitatively or quantitatively. One such approach is meta-analysis, which has over 50 years of literature supporting its usefulness, and is becoming more common in cognitive science. However, changes in technical possibilities by the widespread use of Python and R make it easier to fit more complex models, and even simulate missing data. Here we recommend the use of mega-analyses (based on the aggregation of data sets collected by independent researchers) and hybrid meta- mega-analytic approaches, for cases where raw data are available for some studies. We illustrate the three approaches using a rich test-retest data set of infants’ speech processing as well as synthetic data. We discuss advantages and disadvantages of the three approaches from the viewpoint of a cognitive scientist contemplating their use, and limitations of this article, to be addressed in future work.

## INTRODUCTION

Science is, in principle, a cumulative endeavor, whereby new ideas and results are integrated with previous ones to knit the fabric of knowledge. For most of science’s history, this integration was done mainly via narratives, with newer contributions making textual reference to previous ones. Such a narrative approach has many limitations (see detailed discussion in Cristia et al., in press), of which we highlight here the impossibility of quantitatively integrating results. Some consequences of being unable to produce quantitative syntheses include being unable to detect and interpret small effects, which may not be statistically significant with samples typically used in the field; as well as limited abilities to test for potential moderators. Recent years have seen increased interest in doing cumulative science in ways that address such limitations, typically via results being integrated quantitatively using meta-analyses. Mega-analyses, for their part, involve integrated analyses of raw data collected in multiple sites using a single preprocessing and statistical analysis pipeline. They thus differ from simple analyses in the scope of the data, dealing with more heterogeneous sets since the sites may not have collected data in a coordinated manner; and from meta-analyses in that the raw data are included, rather than group-based statistics. When data are aggregated at the level of the individual (as in this article), this mega-analytic approach can be referred to as parametric individual participant data (IPD) meta-analyses. Mega-analytic approaches require a great deal more effort and coordination with data producers than meta-analyses, and their scope is often more limited than that of meta-analyses by logistical constraints; for instance, it may be impossible to recover raw data from studies published more than 10 years ago. In this article, we explore meta- and mega-analyses together with a third approach that we call “hybrid,” for cases where raw data are available for only some studies. We first describe our chosen case study and why it is useful, and we then turn to a longer introduction to our three approaches (meta-analyses, mega-analyses, and hybrid).

### Study Case: Reliability of Infant Speech Perception Measures

Infant speech perception measures have been central to the development of theories of language acquisition. For example, experimental measures showing that infants’ perception for non-native contrasts varied between 6 and 12 months of age led to the conclusion that phonological acquisition begins as early as this (Werker & Tees, 1984). More recently, these same measures have been argued to be valuable predictors of meaningful individual and group variation in vocabulary size measured concurrently or longitudinally, meta-analyzed in Cristia et al. (2014). One outstanding issue, however, concerns the psychometric properties of such measures, and in particular their reliability. We will call the correlation between two versions of a given measure (such as a test-retest correlation) its *reliability*; and the correlation between that measure and a measure of something else (a potential predictor or predicted variable) its *validity*. Demonstrations within classical test theory suggest that the validity of any measure is bounded by the square root of its reliability (e.g., Michell, 2003). As a result, in an applied context it becomes crucial to know precisely a measure’s reliability, as this directly impacts that measure’s value for any application, including for instance prediction of a potential language learning delay. Even in a basic science context, low reliability could indicate poor instrument properties and a lower likelihood of the result to replicate. For the purposes of the current article, test-retest data sets are useful because they allow us to investigate not only test-retest reliability (which is based on a correlation, whose effect size is from the *r* family), but also the magnitude of effects on the first day of test (whose effect size in the case discussed below is from the *d* family; Rosenthal et al., 1994).

Only two studies have been published reporting on test-retest correlations of infants undergoing the same speech perception measures twice (Cristia et al., 2016; Houston et al., 2007). Since the former paper contains data on the latter, we only discuss the former from now on. Cristia et al. (2016) used a meta-analytic method to combine Houston et al.’s (2007) earlier results with test-retest data collected independently by three research labs (each of which carried out 3–5 studies). The three labs actually did not know the others were also gathering the same kind of data. Meta-analytic methods seem to conceptually fit well the goal of integrating results in such a setting: The authors first estimated the test-retest correlation for each of the 12 studies (13 with Houston et al., 2007), and then derived the overall estimate as the weighted median correlation. Surprisingly, this revealed an overall *r* = .065, with a 95% confidence interval of [−0.12; 0.25]. This null result was not just due to some of the studies providing very small correlation coefficients, but crucially because some of these coefficients were large and negative—which is counterintuitive in the context of test-retest studies. Cristia et al. (2016) made the case that it was appropriate to integrate across all 13 studies because there was no reason to believe that test-retest would yield negative correlations. While this is true, calculating correlations as a measure of test-retest stability within each study and then averaging them is not always equivalent to calculating test-retest stability in behavior taking all studies into account together.

### Alternatives to Meta-analyses: Mega-analyses, IPD Meta-analyses, and Hybrid Approaches

Genetics and neuroimaging research are seeing the emergence of work that discusses the benefits of considering raw data together in what are generally called mega-analyses (e.g., Boedhoe et al., 2019; Costafreda, 2009; Sung et al., 2014). When these data are aggregated at the level of the individual (rather than, e.g., the trial level), we can speak of individual-participant data (IPD) meta-analyses (see chapter 26 of the *Cochrane Handbook*; Higgins et al., 2019; and Verhage et al., 2020, for an example in psychology). In this article, we analyze data aggregated at the level of the individual, so these can be called IPD meta-analyses. However, for ease of expression, we will contrast meta- and mega-analyses. That said, we ask readers to bear in mind that our approach is missing out on the possibility of analyzing even more granular aspects of the data, which is possible in mega-analyses.

Mega-analyses have two key advantages over meta-analyses: homogeneous pre- processing and better handling of variance. As to the first advantage, mega-analyses are preferable because preprocessing steps can be done in a homogeneous fashion, removing this potential source of variance that may be difficult to control for in meta-analyses. Additionally, the second and crucial advantage of mega- over meta-analyses is that structured sources of variance can be better accounted for. This is a point that has been extensively documented in previous methodological literature (e.g., Burke et al., 2017; Legha et al., 2018; Riley et al., 2020), which contrasts typical meta-analyses, which involve two stages (first estimation of effects within studies, then across studies), against IPD meta-analyses, called in this context one-stage models (direct integration of data from several studies in a single model). This point has also been highlighted in the psychological literature, in the context of multi-lab large-scale replications (van Aert, 2020—who also provides a tutorial for conducting IPD meta-analyses). Previous methodological and simulation work has demonstrated that meta-analyses and IPD meta-analyses converge when shared assumptions are met (Burke et al., 2017; Papadimitropoulou et al., 2019). Saliently, Burke et al. (2017) shows that, among the 10 key reasons why one- and two-stage approaches differ, nearly all could be traced back to divergent assumptions, some of which meta-analysts may not have been aware of, and which can be corrected by aligning assumptions across the two ways of analyzing the data.

Regarding these two advantages, as it happens, Cristia et al. (2016) preprocessed all data (except for the published study) in the same way, and thus the first advantage did not apply. The second advantage is relevant for two reasons. First, experiments varied methodologically (some relying on habituation, others on familiarization) and conceptually (e.g., some looked at consonant processing, others at familiar word recognition), and thus they constitute a heterogenous set. Second, among the 13 studies included, 3 were collected on the same group of infants, and yet Cristia and colleagues treated their data as being essentially mutually independent. That said, recent research suggests that infants are relatively inconsistent (DeBolt et al., 2020), to the point that such a data set may not be ideal to assess the effects of structured variance at the level of individuals. In fact, although Cristia et al.’s (2016) data are representative of infant studies in terms of effect and sample size, and thus they are informative for the present study, they also constitute a rather limited data set, which doesn’t allow us, for instance, to assess to what extent different approaches can recover an underlying effect size because we do not know the true underlying effect size for that work. To this end, in this article we also employ simulations, whereby synthetic data are produced with the same structure as would be found in test-retest (infant) studies.

Returning to the question of different approaches to data integration, meta-analyses do have one advantage over mega-analyses, in that more of the literature can be incorporated. That is, after a systematic review reveals what literature could be eligible, the portion that can be included in a meta-analysis includes all the studies that reported the necessary overall effects, as well as any studies for which original authors can provide overall effects. For a mega-analysis, in contrast, one needs access to the raw data. Since only a minority of studies today are published at the same time as data are deposited in scientific archives, this means recruiting the help of the authors of all included studies to retrieve and share the raw data. Even if all authors who are contacted agree, data will be missing for any author who has left the field. We can imagine ways in which this leads to systematic biases in the data, for instance by earlier studies being less likely to be represented than later studies.

Fortunately, advances in data science and statistics provide us with a framework where we can hope to get the best of both worlds. Multiple imputation is a well-developed set of statistical approaches for dealing with missing data (Kleinke et al., 2020; Zhang, 2016). Although it has most commonly been invoked in the context of individual studies, both conceptual insights and, to some extent, statistical tools can also be adopted in the context of integration across studies. In this article, we call this third approach “hybrid,” to represent the fact that it incorporates information at the study level (as in a meta-analysis) and a finer-grained level (as in a mega-analysis).

To our knowledge, this third approach is uncommon when integrating previous literature quantitatively within cognitive science (although we drew inspiration from Fusaroli et al., 2018, who used a statistical approach different from ours). However, there is some previous relevant work on this in the medical literature. A similar proposal has been made by Papadimitropoulou et al. (2019), who called this “pseudo IPD”; we particularly recommend that paper for readers using SPSS or SAS (see their Appendix A for code). That paper analyzed both natural and synthetic data through the example of a systematic review comparing iron blood levels in older adults as a function of Alzheimer diagnosis. They found broadly convergent results for the traditional meta-analysis and an IPD meta-analysis using group statistics to simulate individual participant data to test for group differences.

## THE PRESENT STUDY

Our goal is to illustrate some strengths and weaknesses of alternative cumulative approaches to cognitive experimental research, so that our colleagues within cognitive science have a roadmap for how to do this for their own research goals. To this end, we compare results from three approaches (meta-analysis, mega-analysis, hybrid), paying attention to (a) whether fixed and/or random effects are of interest; (b) whether all, some, or no individual data are available; and (c) whether the focus is experimental effects (i.e., using the first day’s data only) or test-retest reliability (i.e., using the correlations between the first and second days).

We first do this with Cristia et al.’s (2016) data set, which contains our current best estimate for test-retest in infant speech perception tasks. Then, we turn to synthetic test-retest data. Such data sets are informative because they allow us to know for certain what the underlying effect sizes and structured variation are, and thus check the extent to which these effects can be recovered through different analytic approaches.

### A Brief Primer on Test-Retest of Infant Speech Perception

Before proceeding, we give some definitions necessary to understand our explanations. Experts on infant speech perception could skip this section.

In the infant speech perception tasks considered here, infants sit on a caregiver’s lap in a room that either has a frontal visual display, or three displays, one at the front and the others to each side. The visual displays are simply used to allow the child to fixate on the source of an audio track being played, so that the infant’s fixation or looking time can be measured as an index of their attention to the audio track. In all included studies, infants can control the presentation of sound during each trial as follows: The infant’s attention is drawn to the source (by, e.g., a light blinking), and then an audio track begins to play at the same time as a certain image appears on the visual display (e.g., a checkerboard pattern). The audio track, paired with that visual, continue for as long as the child continues to fixate on the image. If the infant looks away for more than a certain threshold (e.g., looks away for more than 2 s), then the audio is interrupted and the visual display is changed back to the attention-getting stimulus. The sequence from when the child fixates on the display, until the time when the audio track stops (because the audio track finished or the infant looked away) constitutes a “trial.”

Studies typically have two phases, an “exposure” phase and a “test” phase, each with multiple trials. During the *exposure* phase, infants listen to an audio track for as long as they fixate on the sound source. Depending on the study design, the same audio track will be repeated in every trial until the infant’s fixation time at the trial level wanes below a certain threshold (e.g., they look less than 50% of the time they looked in the first three trials), or until the infant accumulates a certain listening time. The first design is called “habituation,” and the second “familiarization.” Infants’ subsequent looking time may be affected by this design feature, a point to which we return.

Once the infant has moved on to the *test* phase, trials will typically alternate between two types. One of the types is closer to what the child heard in the exposure phase; for instance, if they were familiarized or habituated with the word *sip*, a “familiar” trial may contain new exemplars of the word *sip*. The other type is relatively more novel; for instance, a “novel” trial in this case will be exemplars of the word *ship*. Looking time is measured during these two trial types, and a preference quotient (PQ) is established as *PQ* = $\frac{{LT}_{N}-{LT}_{F}}{{LT}_{N}+{LT}_{F}}$, where *LT*_*N*_ and *LT*_*F*_ are average looking times for the novel and the familiar trial respectively. PQs vary between −1, indicating the strongest possible preference for familiar trials, and +1, the strongest possible preference for novel trials.

In general, habituation designs are thought to guarantee that infants have exhausted the preference they could have for the familiar stimulus, and thus this design should lead to positive PQs. In contrast, a fixed length of familiarization may not suffice for the infant to fully process the stimulus heard during initial exposure, and thus both familiarity preferences (negative PQs) and novelty preferences (positive PQs) have been observed following familiarization designs (Black & Bergmann, 2017), with familiarity preferences being perhaps more common.

In test-retest designs, we will have a PQ collected on the first day the child sees the task (PQ1). In the case of studies meta-analyzed in Cristia et al. (2016), children were retested after an average of 2 to 7 days, meaning that infants were invited into the lab twice in the same week or in subsequent weeks. During their second visit, infants were faced with the same study they had participated in previously. In the majority of the studies of Cristia et al. (2016), this even meant that they were exposed to the same exact stimuli during the exposure phase, and tested with the exact same stimuli during the test phase; in the exceptional cases, the stimuli in the exposure phase were swapped such that what was relatively novel on the first day was used for the exposure phase on the second day. In any case, this second administration of the infant speech perception task results on a second PQ (PQ2), calculated the same way as in the first day’s test.

The group level performance on each day can be represented with a Cohen’s *d*, defined as the mean of PQs over all infants divided by the standard deviation over all infants. The test-retest correlation is the point biserial correlation between PQ1 (the PQ of the first day) and PQ2 (that of the second day) across all infants. Finally, for some analyses below, we calculate a child-level Cohen’s *d* as the child-level PQ divided by the group-level standard deviation—notice that this is only done so that the resulting effect can be numerically compared with the group-level Cohen’s *d*.

### Modeling

We ran three different analyses: meta-analysis, mega-analysis, and hybrid analysis. The first two were carried out on the natural data on Cristia et al. (2016), and on synthetic data generated for a set of experiments with the same characteristics (Experiment 1 and Experiment 2, respectively). The third analysis was carried out only on Cristia et al.’s (2016) data, since it seemed circular to study the generation of individual data based on group averages that were themselves derived from synthetic data.

This manuscript was produced using RMarkdown (Baumer & Udwin, 2015) and Papaja (Aust & Barth, 2017) on R (R Core Team, 2021) for increased reproducibility. It can be downloaded and reproduced using the data also available from the Open Science Framework, https://osf.io/mk3hx/. For brevity, full model outputs see the Supplemental Materials (https://osf.io/jm649/, Appendix A).

#### Meta-Analysis: No Individual Data Are Known.

For all meta-analyses, we used the rma function in the metafor package (Viechtbauer & Viechtbauer, 2015). The estimator used in all cases was restricted maximum likelihood (REML), and weight was given by the inverse of the variance. For the *infants’ performance on the first day of testing* (i.e., the Cohen’s *d* associated to PQ1), we used only intercept, namely, *y*_*j*_ = *β*_0_ + *u*_0*j*_ + *e*_*j*_, where *y*_*j*_ is the effect size d for the experiment *j*, while *β*_0_ is the intercept (fixed effect), *u*_0*j*_ the deviation to the intercept for each study *j* (random effect), and *e*_*j*_ the residual for each observation. We assume that the true outcomes in the population of studies are unbiased and normally distributed, that is, *u*_0*j*_ ∼ *N*(0, *τ*^2^), and *e*_*j*_ ∼ *N*(0, *v*_*j*_), where *v*_*j*_ are the sampling variances and *τ*^2^ is the variance of true effects in the population (that is, amount of heterogeneity).

In the study of the *test-retest analysis* based on z-transformed correlations *r*_*z*_ between the first and second day’s preference quotients PQ1 and PQ2, we used PQ1 as the only predictor. The model here is *y*_*j*_ = *β*_0_ + *β*_1_*x*_*j*_ + *u*_0*j*_ + *u*_1*j*_*x*_*j*_ + *e*_*j*_, where *y*_*j*_ and *x*_*j*_ are the outcome PQ2 and the predictor PQ1, respectively, for the experiment *j*, *β*_0_ and *β*_1_ are the fixed effects (intercept and slope, respectively), *u*_0*j*_ and *u*_1*j*_ are the random effects associated to intercept and slope, respectively, and *e*_*j*_ the residual for each observation. As in the previous case, *u*_0*j*_ ∼ *N*(0, *τ*^2^), and *e*_*j*_ ∼ *N*(0, *v*_*j*_).^1^

#### Mega-Analysis: Full Individual Data Are Known.

In our mega-analysis, we integrate all individual infants’ data in the same analysis. We used the lmer function from the lme4 package (Bates et al., 2015). Error terms for random effects for mega-analyses and hybrid were estimated with the R library arm (Gelman & Su, 2020).

To assess *infants’ performance on the first day of testing*, we fit a linear mixed model for the child-level Cohen’s *d* on the first day, where fixed effects are absent, and experiment (study) is the only random effect (intercept). We assume random effects are normally distributed; the REML criterion is used to optimize estimates in all models. BOBYQA optimizer was used in all models. In R notation, our model reads *lmer*(*d* ∼ 1 + (1 | *study*)). In general statistical notation, the formula is *y*_*ij*_ = *β*_0_ + *u*_0*j*_ + *e*_*ij*_, where *y*_*ij*_ is the effect size d for observation *i* in the experiment *j*, while *β*_0_ is the intercept (fixed effect), *u*_0*j*_ the deviation to the intercept for each study *j* (random effect), and *e*_*ij*_ the residual for each observation. We incorporate a random intercept per study to account for variation across studies.

One option would have been to use the preference quotient from the first day as the dependent variable (i.e., PQ1). However, this makes it more difficult to directly compare estimates across the meta- and mega-analytic frameworks. Instead, we develop an individual- level measure of Cohen’s *d* by dividing the individual-level PQ1 by the group-level standard deviation of that variable, *d* = $\frac{PQ1}{\sigma \left(PQ1\right)}$. We then interpret the intercept in the mixed model as an index of infants’ performance (in Cohen’s *d*) on the first day of testing.

To assess *test-retest reliability*, the preference quotient of the second day (PQ2) is the dependent variable, and there is one fixed effect for the preference quotient of the first day (PQ1). Again, we included a random intercept and a random slope (modifying the coefficient for PQ1) for study. This is because we expect reliability to vary across studies. In R notation, *lmer*(*PQ*2 ∼ *PQ*1 + (1 + *PQ*1 | *study*)). In general statistical notation, the formula is *y*_*ij*_ = *β*_0_ + *β*_1_*x*_*ij*_ + *u*_0*j*_ + *u*_1*j*_*x*_*ij*_ + *e*_*ij*_, where *y*_*ij*_ and *x*_*ij*_ are the outcome PQ2 and the predictor PQ1, respectively, for the observation *i* in the experiment *j*, *β*_0_ and *β*_1_ are the fixed effects (intercept and slope, respectively), *u*_0*j*_ and *u*_1*j*_ are the random effects associated to intercept and slope, respectively, and *e*_*ij*_ the residual for each observation. In this case, we must interpret the estimate for the fixed effect, which is what indexes the predictive value of the first day’s performance with respect to the performance on the second day, akin to the correlation between these two measures.

Why do we declare a random intercept per experiment as a random factor? For instance, “experiment” could have been a fixed effect, instead of a random effect. We propose that conceptual or methodological factors that are shared across experiments (e.g., stimulus or design decisions) could be declared as fixed effects. In contrast, experiments are best thought of as levels in a random factor, because they are sampling from the space of possibilities, which are partly defined by those conceptual and methodological factors but partly also by other aspects that can be harder to grasp (e.g., time of the year when the studies were collected, characteristics of the particular participants who were tested and which were not logged, etc.).

We can next ask whether *both* a random intercept and a slope are appropriate for the test- retest case. The random intercept captures variation across experiments in PQ2; the slope captures variation in the predictive effect of PQ1 with respect to PQ2. Thus, this random slope captures the intuition that there may be some experiments in which the predictive power of PQ1 is stronger or weaker.

Our fixed and random structure is fairly small but sufficient conceptually and empirically. We encourage other mega-analysts to consider additional random effects that capture structure in the data, such as data coming from different participants, laboratories, or countries. In our case, we did not include random intercept per child or for laboratory because each participant only contributes a single data point for nearly all experiments in the natural data case,^2^ and for all experiments in the synthetic data case (see Hedges et al., 2010, for an alternative approach).

#### Hybrid Analyses: Simulating Individual Data.

Typically, cognitive scientists attempting to do a cumulative analysis may be able to have some individual data released to them by authors, but not all. This will nonetheless leave them with the following sources of information:

- data needed for a group-level analysis (i.e., meta-analysis) for all studies
- raw data needed for an individual-level analysis (i.e., mega-analysis) for *some* studies

We explored multiple approaches to this intermediate case introduced in Supplemental Materials (see Appendix B of the Supplemental Materials). Simulations revealed that generating data for all studies consistent with the group statistics provided results that were convergent with meta- and mega-analytic results, at least in terms of the simple analyses carried out here, in which there are no moderators. Therefore, in what remains, we describe an approach in which all data are generated, which is the extreme case in which no individual-level data are known. Readers can inspect Appendix B of the Supplemental Materials to find simulations that are based on only partially simulated data (i.e., some studies are represented by their real data, and others by simulated data). We encourage readers to replace simulated data with the real data whenever possible, as well as to use the latter to check the extent to which conclusions hold when they are simulated (as we did). In addition, our current approach does not make as efficient use of all information as may be done in more advanced multiple imputation approaches, an issue we return to in the discussion.

For the present analyses, we generated participant-level preference quotients using (a) the mean and standard deviation of the preference quotients for both days and (b) the correlation across individual values for the two. All of these data are known because they are needed for the meta-analytic approach. Readers may also be interested in estimating the influence of moderators (e.g., age, stimuli type). We return to this point in the discussion, when addressing how to extend this approach to more complex questions. We generated participant-level data only for Experiment 1, based on natural data, because Experiment 2 employed simulated data, and it seemed trivial to find convergent results in this case.

The generation procedure is simple: We generate normally-distributed points using the R library simstudy (Goldfeld & Wujciak-Jens, 2020), by providing at the level of the study the number of data points to be generated (number of infants), the average preference quotient for the first and second day, as well as their standard deviations, and the test-retest correlation coefficient. We then filter the resulting generated dataset to only keep it when the parameters of the generated data are sufficiently close to the intended ones (e.g., when the correlation coefficient of the generated data points is within .01 from the intended *r*). This is because generated data may deviate from the requested *r*, but in the context of generating individual-participant data for studies for which this level of data is missing, it is most reasonable to stay as close to the group-level effects as possible. We run 100 iterations of this procedure to focus on results that are reliable. Finally, for each iteration, we fit a model exactly like those used in the mega-analysis section, and we extract estimates for fixed and random effects. Regarding error terms, standard errors have been calculated considering both variation within studies (*SE* of random effects in each simulation) and between studies (*SE* between means of each simulation). Readers can refer to Rubin’s Rules (see, e.g., the pool function in the mice package; Zhang, 2016).

## EXPERIMENT 1: NATURAL DATA

### Data

The original natural data were obtained from https://osf.io/62nrk/. Since all data analyzed here have been described in detail in Cristia et al. (2016), we will simply say that there were a total of 13 experiments, summarized in Table 1.

**Table 1.** Description of experiments.

Keyname	*N*	Age	PQ1	*t* (PQ1)	PQ2	*t* (PQ2)	*r*
f-phrase-LabB	10	6.2 (0.2)	−0.076 (0.125)	−1.92	0.020 (0.117)	0.553	−.174
f-word-LabB	30	6.0 (0.3)	−0.048 (0.170)	−1.537	−0.068 (0.152)	−2.454*	.425*
f-word1-LabC	40	7.6 (0.0)	−0.083 (0.194)	−2.699*	−0.099 (0.124)	−5.05*	−.017
f-word2-LabC	40	10 (0)	−0.030 (0.224)	−0.852	−0.085 (0.119)	−4.545*	−.413*
f-word3-LabC	40	11 (0)	−0.09 (0.231)	−2.458*	−0.134 (0.203)	−4.173*	.052
h-cons-LabA	89	8.7 (1.5)	0.024 (0.219)	1.034	−0.057 (0.257)	−2.087*	−.231*
h-cons-LabB	10	11.1 (0.3)	0.07 (0.227)	0.975	0.104 (0.285)	1.158	−.316
h-vowel-LabA	58	9.6 (1.0)	0.178 (0.225)	6.026*	0.231 (0.205)	8.582*	.313*
h-vowel-LabB	17	5.9 (0.4)	0.138 (0.169)	3.358*	0.210 (0.157)	5.516*	.06
h-word1-LabA	30	6.1 (0.5)	0.221 (0.254)	4.756*	0.298 (0.233)	7.011*	.277
h-word2-LabA	18	9.0 (0.4)	0.23 (0.244)	3.995*	0.202 (0.234)	3.652*	−.171
h-word3-LabA	17	11.6 (0.2)	0.242 (0.248)	4.025*	0.236 (0.258)	3.763*	.358
Houston2007	10	NA (NA)	0.380 (0.143)	8.426*	0.393 (0.160)	7.758*	.699*

*Note*. Keynames indicate whether a familiarization (f) or a habituation procedure (h) was used; what level was targeted (word, vowel, cons[onant], phrase); and which lab data were collected in. When the same experiment was presented to infants of different ages, this is coded in the keyname with a number (i.e., word1, word2, word3). Houston et al. (2007) used a habituation procedure on words. Age mean (*SD*) in months. Preference quotient or each day (PQ1, PQ2) with its SD as well as *t* against no preference, separately for each day (1, 2). *r* = correlation across days.

### Results

#### Meta-Analysis: No Individual Data Are Known.

We first fit a random-effect meta-regression on the *infants’ performance on the first day of testing* (i.e., the Cohen’s *d* associated to PQ1). The overall effect size in the first day of testing, collapsing across studies, was estimated at Cohen’s *d* intercept = 0.35 (95% CI: [−0.04, 0.74]), *SE* = 0.20, *z* = 1.74, *p* = .08. This trend is broadly consistent with a preference quotient greater than zero in the first day of testing.

**Figure 1.**
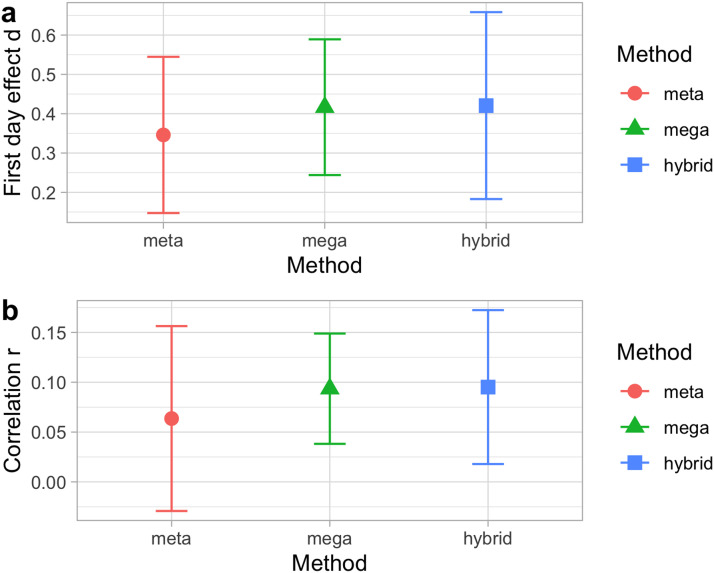
**Fixed effects in the analysis of (a) effect size in the first day of testing and (b) the test-retest correlation, according to three estimation methods: A meta-analysis (meta); a regression with the true individual-level data (mega); and a regression where all individual-level data points have been generated (hybrid).** Error bars indicate standard errors. In the hybrid analysis (hybrid), standard errors have been calculated considering both variation within studies (*SE* of fixed effects in each simulation) and between studies (*SE* between means of each simulation).

For the *test-retest analysis*, as concluded in the original study, the overall test-retest correlation across studies was not different from zero, *z*-transformed *r* of *r*_z_ = .06 (95% CI: [−0.12, 0.25]), *SE* = 0.09, *z* = 0.68, *p* = .49. This is consistent with an interpretation whereby there is no stability in infant performance across test and retest. Results for meta-analytic outcomes for both first day performance and test-retest are plotted in Figure 1, together with the outcomes according to the alternative methods, which are presented in subsequent sections.

**Figure 2.**
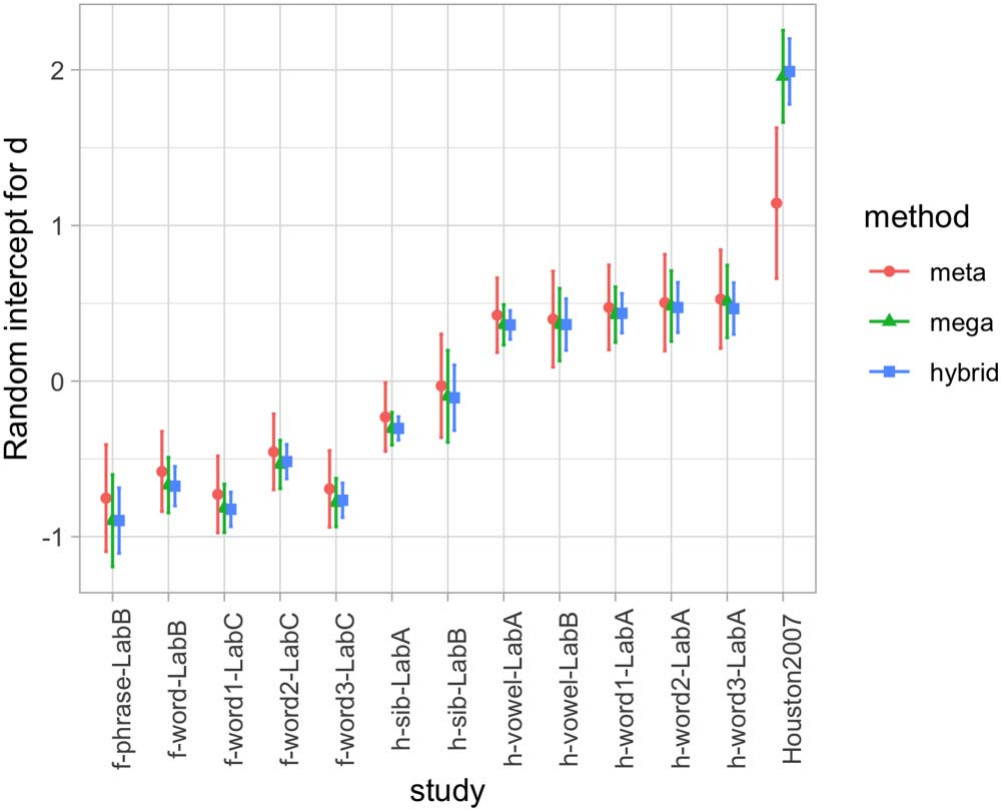
**Random intercepts per study in the analysis of effects in the first day of testing, representing deviations from the fixed effects in Figure 1a, according to three estimation methods: A meta-analysis (meta); a regression with the true individual-level data (mega); and a regression where all individual-level data points have been generated (hybrid).** Error bars indicate standard errors. In the hybrid analysis (hybrid), standard errors have been calculated considering both variation within studies (*SE* of random effects in each simulation) and between studies (*SE* between means of each simulation).

**Figure 3.**
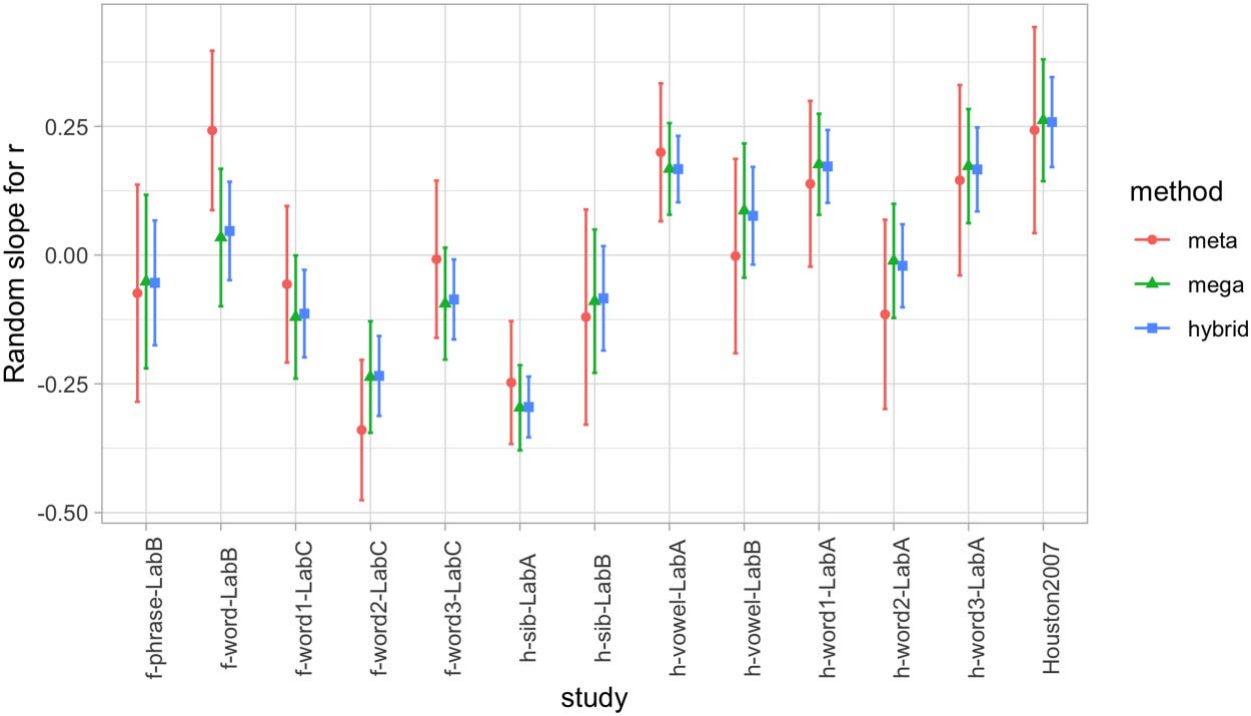
**Random slopes per study in the analysis of test-retest correlation, representing deviations from the fixed effects in Figure 1b, according to three estimation methods: A meta-analysis (meta); a regression with the true individual-level data (mega); and a regression where all individual-level data points have been generated (hybrid).** Error bars indicate standard errors. In the hybrid analysis (hybrid), standard errors have been calculated considering both variation within studies (*SE* of random effects in each simulation) and between studies (*SE* between means of each simulation).

In both cases, however, there is wide variation across the different studies, as evident in Table 1. Notice that PQ1 and the corresponding Cohen’s *d* can be positive or negative; and that *r*’s also vary in strength and direction across studies. Thus, the choice of fitting a random-effects meta-analysis, made on a conceptual ground (because these studies are not pure replications), is further supported by the data. We therefore extracted the variation in random effects for each of the two analyses. Figure 2 shows the random intercepts per study for the analysis of PQ1, and Figure 3 the random slopes per study in the analysis of the correlation between PQ1 and PQ2. In each case, we also show the corresponding values according to our other methods, which are introduced in subsequent sections. Note that Figure 3 shows the random intercepts in a random effect meta-analysis on the z-transformed correlation, and thus these intercepts are conceptually akin to a slope in a regression predicting PQ2 from PQ1.^3^ From Figures 2 and 3, we can see how the significant variation across studies shown in Table 1 is reflected in the range of random effects, both for intercept (Figure 2) and for slope (Figure 3).

#### Mega-Analysis: Full Individual Data Are Known.

*Infants’ performance on the first day of testing*: The estimate for the Cohen’s *d* on day 1 was: estimate = 0.42 (95% CI: [−0.06, 0.91]), *SE* = 0.24, *t* = 1.77, *p* = .10 (Figure 1a). The estimate is positive, consistent with a trend for PQ1 being positive when all studies are considered together. The random effects per experiment accounted for 41% of the variance unexplained by the fixed effects. The distribution of random effects can be seen in Figure 2.

*Test-retest reliability*: The estimate for day 2’s preference quotient predicting day 1’s PQ was: (a) Intercept: estimate = 0.07 (95% CI: [−0.06, 0.26]), *SE* = 0.04, *t* = 1.63, *p* = .13, and (b) slope: estimate = 0.10, *SE* = 0.08, *t* = 1.23, *p* = .24. This value is equivalent to a correlation coefficient of *r* = .09 (Figure 1b). Thus, we replicate the main conclusion of no global predictive value of the second day’s preference quotient from the first day’s one. However, the structure of random effects associated to the experiment sheds light on this correlation. For example, it can be seen from Figure 2 that experiments such as h-vowel-LabA and Houston et al. (2007) have a significantly positive random effect for their slopes (which should be summed to the also positive fixed effect in order to obtain the correlation for each study), consistently across methods. Random intercepts per experiment accounted for 19% of the variance, random slopes for 42%; as a result, 39% of the variance was left unexplained. The distribution of random effects can be seen in Figure 3.

#### Hybrid Analyses: Simulating Individual Data.

*Infants’ performance on the first day of testing*: The median (ranges) across 100 iterations were: estimate = 0.42 (range 0.28–0.56), *SE* = 0.24 (range 0.20–0.30), *t* = 1.69 (range 1.03–2.49), *p* = .12 (range .03–.32) (Figure 1a). The distribution of random effects can be seen in Figure 2.

*Test-retest reliability*: The intercept value across 100 iterations was: estimate = 0.07 (range 0.03–0.10), *SE* = 0.04 (range 0.03–0.05), *t* = 1.65 (range 0.88–2.14), *p* = .13 (range .05–.40); and for the slope, estimate = 0.09 (range 0.06–0.15), *SE* = 0.08 (range 0.06–0.09), *t* = 1.17 (range 0.78–1.71), *p* = .27 (range .12–.46) (Figure 1b). The distribution of random effects can be seen in Figure 3.

### Discussion

#### Comparison of Results Pertaining to Fixed Effects.

As seen in the top panel of Figure 1, the estimate for *infants’ performance on the first day of testing* is slightly higher when using mega-analyses on real or simulated data (i.e., mega and hybrid) as opposed to when using meta-analyses, and the error bars for mega-analyses are shorter than those for meta-analysis and hybrid. Nonetheless, there is a great deal of overlap across all three distributions.

The lower panel of Figure 1 shows that estimations are also numerically higher for mega- analysis and hybrid than meta-analysis for *test-retest reliability*, and the error bars were shorter for mega-analysis than meta-analysis or hybrid. None of the three analyses achieved the alpha .05 significance level, and thus it appears that the conclusion that infants’ performance on the first day does not predict the second day’s drawn by Cristia et al. (2016) held across all three analyses, at least in terms of the overall estimates collapsing across studies.

#### Comparison of Results Pertaining to Random Effects.

Our case study was useful because there was systematic variation across different experiments, captured here through random effects. With one exception (corresponding to Houston et al., 2007, which has a much larger effect than the other studies^4^), all three methods provide essentially convergent evidence on the variation across experiments when looking at *infants’ performance on the first day of testing* (Figure 2).

Results on the random effects in the analysis of *test-retest reliability* show a great deal less convergence across the three methods. A majority of the estimates emerging from mega-analysis are within the range of the estimate ± one standard error extracted from meta-analyses, and all three methods tend to converge in terms of whether their estimates for a given study are positive or negative, but clearly less well than in the analysis of infants’ performance on the first day. This probably happens because the mega-analysis and hybrid approaches depart from the meta-analysis in that the meta-analysis has error terms for the *correlation*, whereas the mega-analysis and hybrid approaches have error terms for intercepts and slopes. Thus, this is a case where the divergence in results relates to divergent assumptions across statistical approaches (Burke et al., 2017).

## EXPERIMENT 2: SYNTHETIC DATA

The previous experiment is informative because it represents the results of our proposed approaches when faced with a natural data set. However, it seemed insufficient in our view for two important reasons. First, in natural data we do not know the true underlying fixed and random effects’ sizes. Second, it is difficult to cover a wide range of possibilities with natural data, including particularly large effect sizes—which are seldom observed among infants this young (Bergmann et al., 2018). In this experiment, we do not employ the hybrid approach because it would have meant generating data that had been already generated (even using the same package)—making any discovery that these two converge trivial. We present here results from runs assuming the same number of participants per study; see Appendix C of the Supplemental Materials for simulations relaxing this assumption.

### Data

In this experiment, we generate synthetic data for 10 simulated studies, each consisting of 100 participants, using the R library simstudy (Goldfeld & Wujciak-Jens, 2020). We systematically and independently vary (i) The preference quotient for the first day’s test PQ1 (which determines our effect size Cohen’s *d* for PQ1) and that for the second day PQ2, (ii) The test-retest correlation in performance across days *r*. We focus on a homogeneus data set, where all 10 studies are replications with the same parameters. We return to this choice in the discussion. Simulations were made for values of PQ1 = 0, .30, and .60, and *r* = .15, .50, and .85. The standard deviation for PQ1 and PQ2 inside each study was set as *σ*(*PQ*) = .15 in all cases. Therefore, the values for effect size were *d* = 0, 2, and 4. A normally distributed standard deviation, different for each of the 10 simulated studies, was added to both parameters, *σ*(*d*) = 0.10, and *σ*(*r*) = 0.20, 0.15, and 0.06, respectively. This added deviation should be detected as random effect per study.

### Results

**Figure 4.**
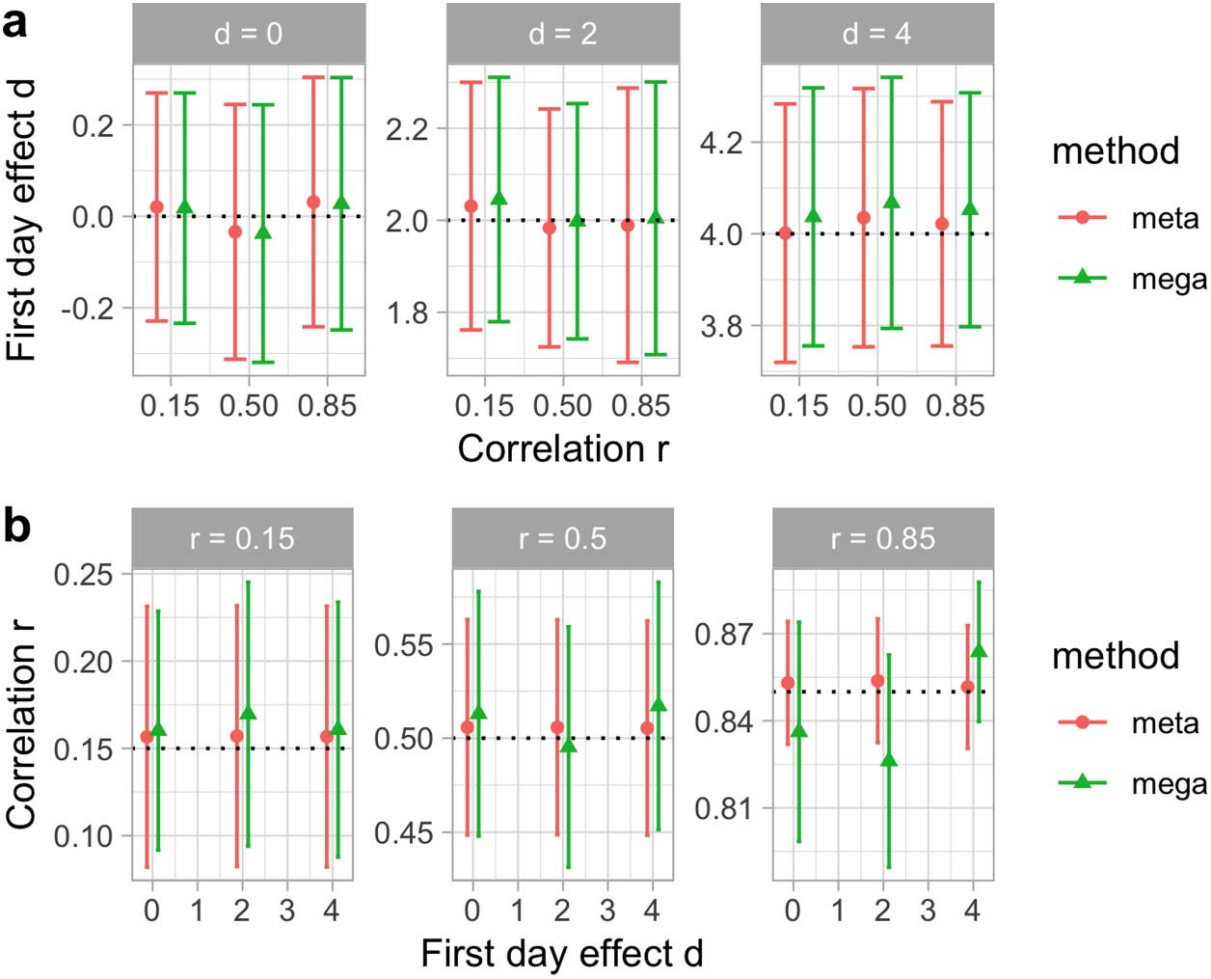
**Fixed effects for synthetic data in the analysis of effect size in the first day of testing (a) and of the correlation size across testing days (b), according to two estimation methods: A meta-analysis (meta); a regression with the true individual-level data (mega).** In (a), we plot calculated *d* as a function of true correlation *r* in the data generation, and each horizontal panel represents different values of true effect size *d*. In (b), we plot calculated *r* as a function of true effect size *d* in data generation, and each horizontal panel represents different values of true correlation *r*. Error bars represent standard errors for the fitted values. Horizontal dotted lines represent the true value of the parameter (*d* at the top, *r* at the bottom).

Figure 4 shows the results of our simulations for homogeneous data (akin to habituation experiments), where the 10 simulated studies correspond to replications. As expected, both mega- and meta-analysis can detect the values for the first day of testing *d*, and for the test-retest correlation *r*. The values for both analyses are practically the same, and their confidence intervals overlap almost exactly.

**Figure 5.**
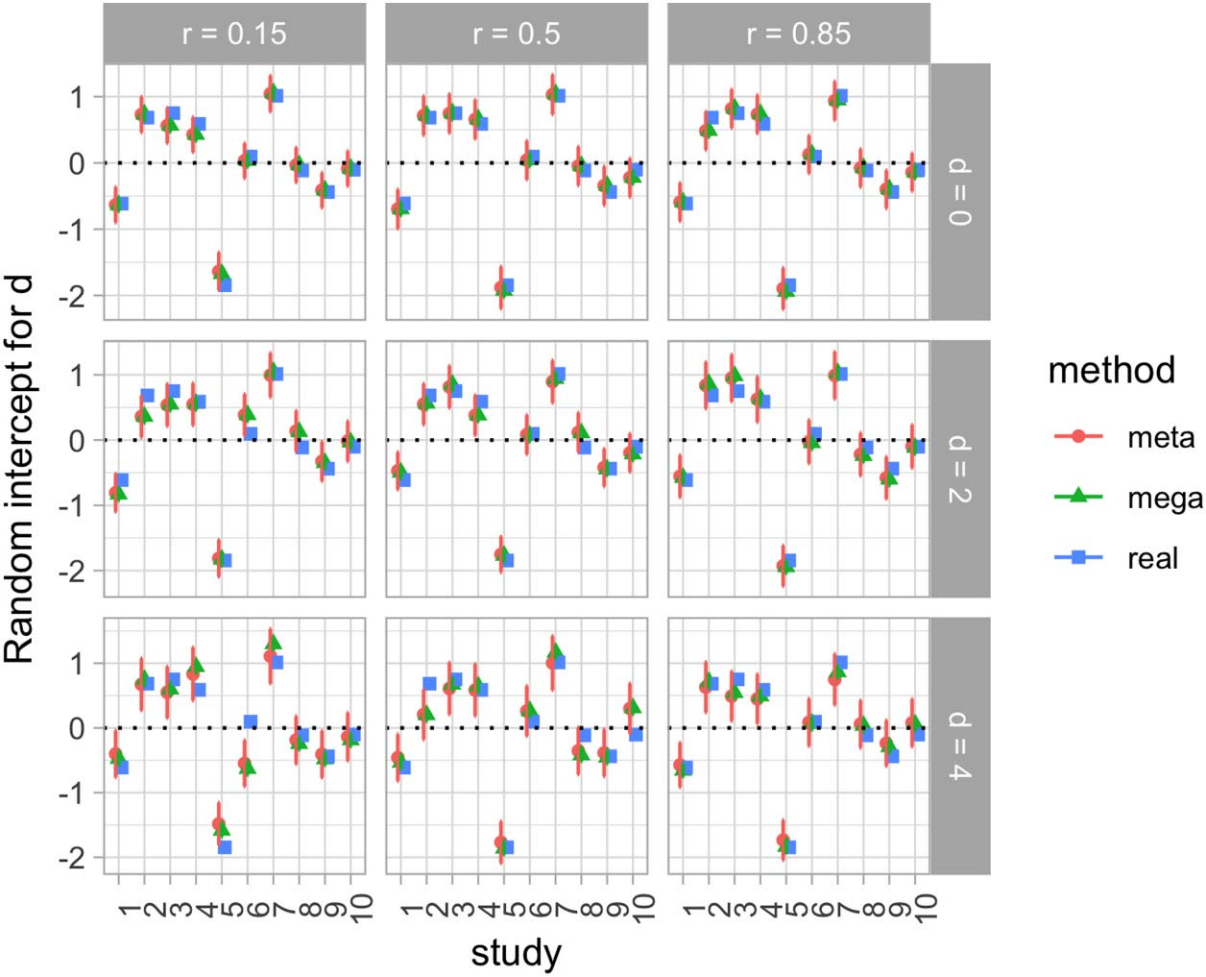
**Random intercepts per study for synthetic data in the analysis of effects in the first day of testing, according to two estimation methods: A meta-analysis (meta); a regression with the true individual-level data (mega).** In each panel, we plot calculated *d* as a function of the true values of *d* and *r* in the data generation. Error bars represent standard errors for the fitted values.

Figure 5 shows random intercepts per study for the first day effect sizes (Cohen’s *d* for PQ1) in the synthetic data set, calculated with meta- and mega-analyses, and compared with the real random variation generated for each of the 10 studies. We can see how both meta- and mega-analyses show a good match with the true values.

**Figure 6.**
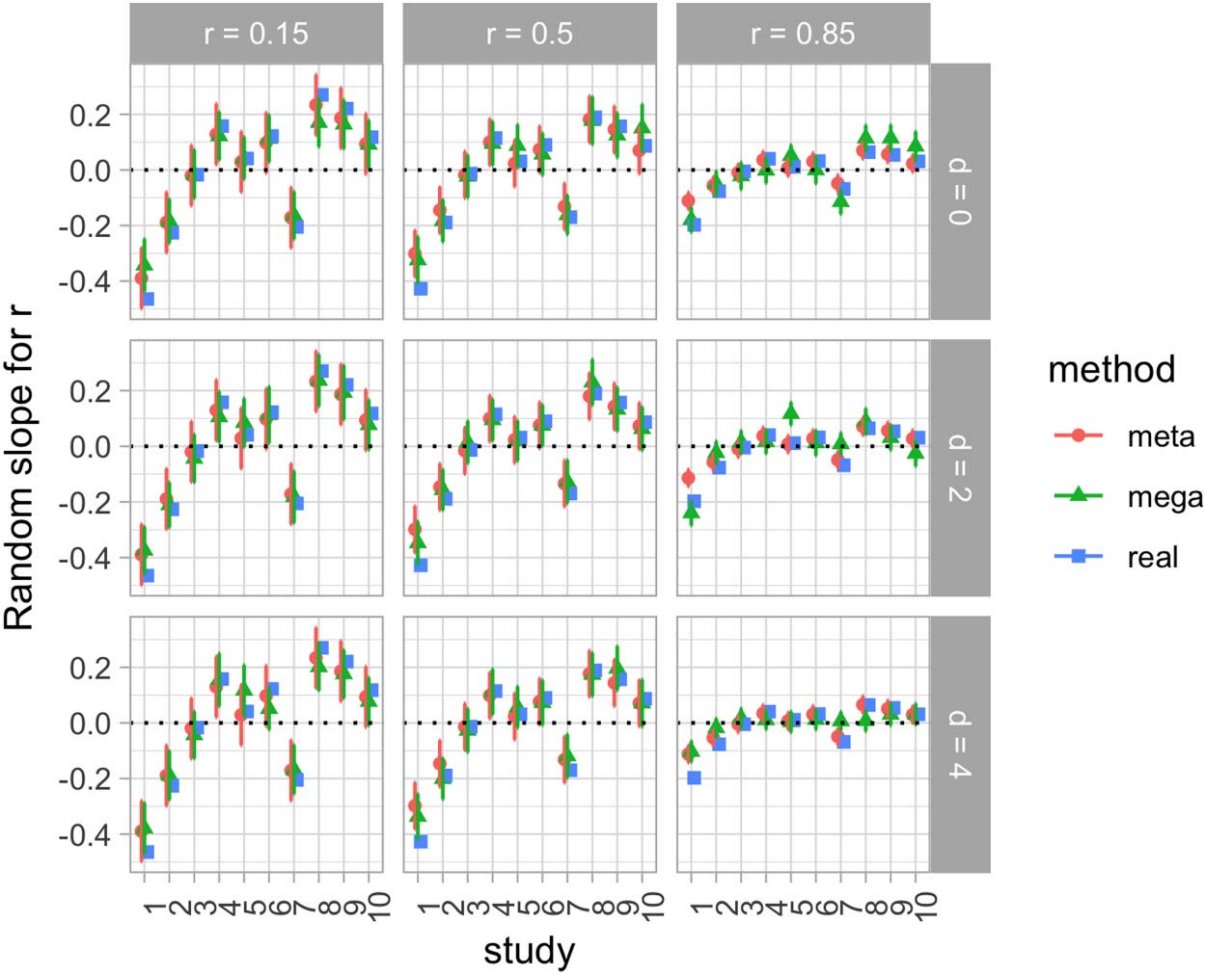
**Random slopes per study for synthetic data in the analysis of the correlation between days of testing, according to two estimation methods: A meta-analysis (meta); a regression with the true individual-level data (mega).** In each panel, we plot calculated d as a function of the true values of *d* and *r* in the data generation. Error bars represent standard errors for the fitted values.

Figure 6 shows random slopes per study for the test-retest correlation in the synthetic data set, calculated with meta- and mega-analyses, and compared with the real random variation generated for each of the 10 studies. We observe a quite reasonable match between both meta- and mega-analyses, and also a good match with the real values in most cases.

### Discussion

Mega-analysis and meta-analysis on the bivariate normally distributed synthetic data generated for Experiment 2 leads to similar conclusions to the natural data analyzed in Experiment 1. Specifically, both fixed and random effects estimated across these two methods are remarkably similar. Experiment 2 goes beyond the insights gleaned from Experiment 1 in that we can now verify that both approaches were able to recover fixed effects of a wide range of sizes.

## GENERAL DISCUSSION

In this article, we ran two experiments to compare three approaches to cumulating data across studies. Experiment 1 used a rich data set that contains data from infants who were tested twice in a short period of time, allowing us to look at main effects in the first day of testing, test-retest correlations, and random effects for both of these. Experiment 2 employed synthetic data with a similar structure, but more extreme effects both for the first day of testing and the test-retest correlation, with the added benefit of knowing what the true fixed and random effects were by setting them as parameters during data generation. We compared traditional meta-analyses with mega-analyses in which all individual participants’ data are known (akin to IPD meta-analyses) in both experiments, and in Experiment 1 additionally with a hybrid approach in which missing individual data are generated using study-level information. Taking all of our results together, we draw the following key messages that we would like cognitive scientists to bear in mind when attempting a cumulative approach to experimental data.

First, if the main research goal is to establish the relevance of fixed effects, one could conclude from the current analyses that a meta-analytic approach is sufficient. In fact, it yields essentially the same results as the mega-analytic and hybrid approach, and it requires less effort overall. Another advantage of meta-analyses is that, by having a wider coverage, they can be particularly informative in the context of questions regarding potential literature-level biases (including selective reporting and/or publication).

That said, we do hope that our first take-home message will not be the main one of relevance to our readers. It is seldom the case that a research question is so simple that one can settle on an answer given the fixed effects’ distribution only. Moreover, meta-analytic analyses, as well as the kind of mega-analytic analyses we performed here, are limited in how informative they are, precisely because of the fact that they remain at a superficial (group) level of description.

Therefore, our second and key take-home message is that the hybrid approach is extremely promising, since it shows very similar results to a mega-analytic one, without requiring all data to be available. Although we fit a simple mega-analysis in the present article, this can be complexified, for example, by adding moderators and interactions. This logic can be easily extended to hybrid approaches, giving more power to future realistic hybrid approaches. We find that this is encouraging, as it seems unlikely that all data will be available for every research question. Additionally, we can always get the best of both worlds by using the meta-analytic approach to shed light on potential literature-level biases, but a hybrid approach for digging deeper into specific effects and relationships.

We have aimed for the current article to be as simple as possible, in order to highlight the fact that the hybrid approach is within most readers’ toolkit already. Specifically, we have used a handy and easy-to-use R package to simulate data (simstudy); and another to analyze it (lme4).

This article is shared in reproducible format on the Open Science Framework under an Apache license, and we truly hope that readers will borrow from our code.

For readers who would like to learn more about these different methods, we can recommend more advanced statistical approaches, which are currently found in applied statistics venues. In particular, Burke et al. (2017) provides an excellent overview of ways in which traditional meta-analyses diverge from IPD meta-analyses. Additionally, Riley et al. (2020) discusses when IPD should be used rather than traditional meta-analyses, with a focus on how to generalize insights gleaned from one or the other method to individual participants outside of the data (i.e., out of sample generalization).

### Potential Limitations

Given our main goal of providing an entry point to this method for cognitive scientists with a wide range of levels of statistical expertise, we have not covered all possible use cases and approaches. We close our discussion with four salient limitations.

The first limitation pertains to the moderators or independent variables: We only explored one quantitative moderator for which we had quite a bit of information (predicting day 2 performance from day 1’s having group-level *r*). In trying to keep our explanation simple, we did not include other fixed effects, such as infant age or the two subclasses of studies (habituation and familiarization). Additionally, our proposed hybrid approach may be less useful in cases in which authors do not report group-level parameters informing the strength of a relationship with a quantitative or qualitative moderator. For instance, we may have reason to believe that the experimenter’s sex affects performance (Brown et al., 2014), but authors seldom report how many participants were tested by males versus females and what the group effects separating these two subgroups were. This is indeed a case where true mega-analyses, involving the collaboration of all data producers, can be more informative than both meta-analysis and hybrid approaches.

A second limitation is that our approaches above used simple formulas to fit the data, with computer packages that can be described as frequentist. Many additional approaches could be adopted, and we hope readers of this article will explore some of them. For example, we use a rather simplistic approach to simulate data, but there exist solutions for multiple imputation that take into account not only broad statistics (like the ones used here, mean and standard deviation), but also the distribution of other data points and even the actual model that one is attempting to fit (Kleinke et al., 2020; Zhang, 2016). To give another example, a set of equations could be defined at the group and individual levels, to be fitted jointly, similarly to Fusaroli et al.’s (2018) approach. Alternatively, a Bayesian approach could be employed, with the subsets of the data for which only group statistics are available providing information to be encoded as priors, and the data for individual participants to fit the model and generate posteriors. What is more, as data availability becomes prevalent, and collaboration across laboratories become wide-spread, our mega-analytic approach here, which is essentially an IPD meta-analysis, could be abandoned for a richer take on the data, using individual trials or even finer-grained temporal representations of results. According to Haines et al. (2020), if such a move were accompanied by more informed, generative models, this would also result in more informative analyses both in terms of psychological theories and the reliable measurement of individual performance. We would look forward to this article quickly becoming outdated due to an increased prevalence of both greater data sharing and more informed approaches to cognitive experimental data.

Third, our simulations assumed individual data were missing at random. This represents the case in which authors’ failure to contribute data is unrelated to the data itself. Simulating all of the data led to essentially the same results as having access to all the real individual-level data (see Appendix B of the Supplemental Materials), leaving us with no room to study the effects of non-random missing data. We can imagine two radically different scenarios, leading to potentially different effects: perhaps authors with larger effects may be more likely to share; or authors with null effects may do so. As the techniques illustrated here become more prevalent, we hope this question is revisited, at the very least studying whether individual data availability may reflect something similar to publication biases. Nonetheless, if our conclusion that missingness has small effects is upheld, such an analysis may be most interesting in terms of sociological and meta-scientific interpretations.

Fourth, our main goal was to illustrate how these methods can be employed, and for this we relied on extant methodological work being carried out mainly in the field of statistics applied to medicine. To make the most of cumulative approaches to cognition, however, it may be necessary to engage in more specific methodological work (for instance, not just showing convergence in estimates but more precisely pinpointing bias, coverage and error) using data sets like those available in our field. This does not mean we need to redo all methodological work again for a cognitive science audience, but at least we should consider in what ways advice from other fields needs to be adapted. For instance, Dewidar et al. (2021) provide a clear and straightforward step-by-step approach to performing IPD meta-analyses. While the early phases require little adaptation (processing, replicating, imputing, and merging data), the final stage of “evaluating data heterogeneity” will likely be the most challenging to us because cognitive measures are seldom standardized, particularly across sites. To give a specific example, Dewidar et al. (2021) explain how they corrected for hemoglobin levels for studies that sampled from populations residing over 1,000 m above sea level, thus rendering hemoglobin levels more comparable across studies. Extrapolating this to our running case study, we have little knowledge of how population-level factors affect infants’ novelty preferences, and thus this level of control for between-study heterogeneity seems difficult to achieve at present. And considering other domains of cognitive science, it would be necessary to reevaluate the promise of approaches like the ones we have explored here for more variable outcomes (e.g., different measures of vocabulary size, or different scales of anxiety).

## CONCLUSION

To conclude, cumulative approaches are fundamental for cognitive science to establish conclusions on a solid empirical base, and develop theories that make relevant contact with these results. Meta-analyses are an important step in this direction. However, in 2021, the ease with which we can program simulations and analyses, together with changes in the prevalence of data sharing can help us go well beyond meta-analyses. Previous work has already highlighted the advantages of IPD meta-analyses over traditional meta-analyses, saliently the more efficient handling of structured variance (e.g., Verhage et al., 2020). We hope that the present study illustrates the ease with which we can all produce more informative reports using mega-analyses or hybrid approaches.

## FUNDING INFORMATION

AC, Agence Nationale de la Recherche, Award ID: ANR-16-DATA-0004 ACLEW, ANR-14-CE30-0003 MechELex, ANR-17-EURE-0017. AC, James S. McDonnell Foundation (https://dx.doi.org/10.13039/100000913), Award ID: Understanding Human Cognition Scholar Award.

## AUTHOR CONTRIBUTIONS

EK: Conceptualization: Equal; Data curation: Equal; Formal analysis: Lead; Investigation: Equal; Methodology: Lead; Visualization: Lead; Writing – original draft: Equal; Writing – review & editing: Equal. AC: Conceptualization: Lead; Data curation: Equal; Formal analysis: Supporting; Funding acquisition: Lead; Investigation: Equal; Methodology: Supporting; Project administration: Lead; Resources: Lead; Supervision: Lead; Visualization: Supporting; Writing – original draft: Lead; Writing – review & editing: Equal.

## Notes

^1^ Notice we practically reproduced the original analyses. The only difference is that we are using the REML estimator instead of the DerSimonian-Laird estimator, as in Cristia et al. (2016). This has been done for making our analysis more comparable with the mega-analysis in the next section. Results are similar with both estimators. See the Supplemental Materials (https://osf.io/jm649/, page 2) for results with the DerSimonian-Laird estimator.^2^ Although LabC tested the same infants at three ages, preliminary analyses revealed that the contribution of random slopes per child was negligible, and we removed them (see Appendix A of the Supplemental Materials). Including random effects per lab would capture the non-independence of data coming from infants tested in the same lab and likely sampled from the same community. However, given that there are only three labs and experiments and infants are fully nested within labs, variance attribution would have been difficult in the present case.^3^ Strictly speaking, we are plotting here the *β* coefficient instead of the Pearson’s correlation *r* for both the mega-analysis and hybrid. The equivalence between these parameters is *β* = $\frac{\sigma_{PQ2}}{\sigma_{PQ1}}$ *r*. Since the values for both *σ* are similar for all experiments, these different measures are comparable considering the dispersions involved. For the case of meta-analysis, we did transform the z-transformed correlation coefficient back into *r*: *r* = tanh(*r*_*z*_) in the plots, for comparability.^4^ It is beyond the scope of the present article to comment on the extent to which the other 12 studies can be seen as replications of this initial study, but we encourage readers interested in the topic to consider Mathur and VanderWeele (2019, 2020) for useful metrics.

## Supplemental Materials

https://osf.io/mk3hx/?view_only=e81ae85d42f04565a8a726de221e2c2a

## References

[bib1] Aust, F., & Barth, M. (2017). Papaja: Prepare reproducible APA journal articles with R Markdown. R package version 0.1.0.9997. GitHub. https://github.com/crsh/papaja

[bib2] Bates, D., Mächler, M., Bolker, B., & Walker, S. (2015). Fitting linear mixed-effects models Using lme4. Journal of Statistical Software, 67(1). 10.18637/jss.v067.i01

[bib3] Baumer, B., & Udwin, D. (2015). R markdown. Wiley Interdisciplinary Reviews: Computational Statistics, 7(3), 167–177. 10.1002/wics.1348

[bib4] Bergmann, C., Tsuji, S., Piccinini, P. E., Lewis, M. L., Braginsky, M., Frank, M. C., & Cristia, A. (2018). Promoting replicability in developmental research through meta-analyses: Insights from language acquisition research. Child Development, 89(6), 1996–2009. https://doi.org/10.1111/cdev.13079, PubMed: 29736962 29736962 10.1111/cdev.13079 PMC6282795

[bib5] Black, A., & Bergmann, C. (2017). Quantifying infants’ statistical word segmentation: A meta-analysis. In G. Gunzelmann, A. Howes, T. Tenbrink, & E. Davelaar (Eds.), Proceedings of the 39th Annual Meeting of the Cognitive Science Society (pp. 124–129). Cognitive Science Society.

[bib6] Boedhoe, P. S., Heymans, M. W., Schmaal, L., Abe, Y., Alonso, P., Ameis, S. H., Anticevic, A., Arnold, P. D., Batistuzzo, M. C., Benedetti, F., Beucke, J. C., Bollettini, I., Bose, A., Brem, S., Calvo, A., Calvo, R., Cheng, Y., Cho, K. I. K., Ciullo, V., Dallaspezia, S., … Twisk, J. W. R. (2019). An empirical comparison of meta-and mega-analysis with data from the enigma obsessive-compulsive disorder working group. Frontiers in Neuroinformatics, 12, Article 102. https://doi.org/10.3389/fninf.2018.00102, PubMed: 30670959 30670959 10.3389/fninf.2018.00102 PMC6331928

[bib7] Brown, S. D., Furrow, D., Hill, D. F., Gable, J. C., Porter, L. P., & Jacobs, W. J. (2014). A duty to describe: Better the devil you know than the devil you don’t. Perspectives on Psychological Science, 9(6), 626–640. https://doi.org/10.1177/1745691614551749, PubMed: 26186113 26186113 10.1177/1745691614551749

[bib8] Burke, D. L., Ensor, J., & Riley, R. D. (2017). Meta-analysis using individual participant data: One-stage and two-stage approaches, and why they may differ. Statistics in Medicine, 36(5), 855–875. https://doi.org/10.1002/sim.7141, PubMed: 27747915 27747915 10.1002/sim.7141 PMC5297998

[bib9] Costafreda, S. G. (2009). Pooling fMRI data: Meta-analysis, mega-analysis and multi-center studies. Frontiers in Neuroinformatics, 3, 33–41. https://doi.org/10.3389/neuro.11.033.2009, PubMed: 19826498 19826498 10.3389/neuro.11.033.2009 PMC2759345

[bib10] Cristia, A., Seidl, A., Junge, C., Soderstrom, M., & Hagoort, P. (2014). Predicting individual variation in language from infant speech perception measures. Child Development, 85(4), 1330–1345. https://doi.org/10.1111/cdev.12193, PubMed: 24320112 24320112 10.1111/cdev.12193

[bib11] Cristia, A., Seidl, A., Singh, L., & Houston, D. (2016). Test-retest reliability in infant speech perception tasks. Infancy, 21(5), 648–667. https://osf.io/62nrk/. 10.1111/infa.12127

[bib12] Cristia, A., Tsuji, S., & Bergmann, C. (in press). A meta-analytic approach to evaluating the explanatory adequacy of theories. Metapscyhology. 10.31219/osf.io/83kg2

[bib13] DeBolt, M. C., Rhemtulla, M., & Oakes, L. M. (2020). Robust data and power in infant research: A case study of the effect of number of infants and number of trials in visual preference procedures. Infancy, 25(4), 393–419. https://doi.org/10.1111/infa.12337, PubMed: 32744759 32744759 10.1111/infa.12337

[bib14] Dewidar, O., Riddle, A., Ghogomu, E., Hossain, A., Arora, P., Bhutta, Z. A., Black, R. E., Cousens, S., Gaffey, M. F., Mathew, C., Trawin, J., Tugwell, P., Welch, V., & Wells, G. A. (2021). PRIME-ipd series part 1. The prime-ipd tool promoted verification and standardization of study datasets retrieved for ipd meta-analysis. Journal of Clinical Epidemiology, 136, 227–234. https://doi.org/10.1016/j.jclinepi.2021.05.007, PubMed: 34044099 34044099 10.1016/j.jclinepi.2021.05.007 PMC8442853

[bib15] Fusaroli, R., Weed, E., Lambrechts, A., Bowler, D. M., & Gaigg, S. B. (2018). Towards a cumulative science of prosody in ASD [Poster presentation]. INSAR. https://insar.confex.com/insar/2018/webprogram/Paper26404.html

[bib16] Gelman, A., & Su, Y.-S. (2020). Arm: Data analysis using regression and multilevel/hierarchical models. CRAN. https://CRAN.R-project.org/package=arm

[bib17] Goldfeld, K., & Wujciak-Jens, J. (2020). Simstudy: Simulation of study data. CRAN. https://CRAN.R-project.org/package=simstudy

[bib18] Haines, N., Kvam, P. D., Irving, L. H., Smith, C., Beauchaine, T. P., Pitt, M. A., Ahn, W.-Y., & Turner, B. (2020). Learning from the reliability paradox: How theoretically informed generative models can advance the social, behavioral, and brain sciences. PsyArXiv. https://psyarxiv.com/xr7y3/. 10.31234/osf.io/xr7y3 40232753

[bib19] Hedges, L. V., Tipton, E., & Johnson, M. C. (2010). Robust variance estimation in meta- regression with dependent effect size estimates. Research Synthesis Methods, 1(1), 39–65. https://doi.org/10.1002/jrsm.5, PubMed: 26056092 26056092 10.1002/jrsm.5

[bib20] Higgins, J. P., Thomas, J., Chandler, J., Cumpston, M., Li, T., Page, M. J., & Welch, V. A. (2019). Cochrane handbook for systematic reviews of interventions. John Wiley & Sons. 10.1002/9781119536604 PMC10284251 31643080

[bib21] Houston, D., Horn, D. L., Qi, R., Ting, J. Y., & Gao, S. (2007). Assessing speech discrimination in individual infants. Infancy, 12, 119–145. https://doi.org/10.1111/j.1532-7078.2007.tb00237.x, PubMed: 33412746 33412746 10.1111/j.1532-7078.2007.tb00237.x

[bib22] Kleinke, K., Reinecke, J., Salfrán, D., & Spiess, M. (2020). Applied multiple imputation. Springer. 10.1007/978-3-030-38164-6

[bib23] Legha, A., Riley, R. D., Ensor, J., Snell, K. I., Morris, T. P., & Burke, D. L. (2018). Individual participant data meta-analysis of continuous outcomes: A comparison of approaches for specifying and estimating one-stage models. Statistics in Medicine, 37(29), 4404–4420. https://doi.org/10.1002/sim.7930, PubMed: 30101507 30101507 10.1002/sim.7930 PMC6283045

[bib24] Mathur, M. B., & VanderWeele, T. J. (2019). Challenges and suggestions for defining replication “success” when effects may be heterogeneous: Comment on Hedges and Schauer (2019). Psychological Methods, 24(5), 571–575. https://doi.org/10.1037/met0000223, PubMed: 31580141 31580141 10.1037/met0000223 PMC6779319

[bib25] Mathur, M. B., & VanderWeele, T. J. (2020). New statistical metrics for multisite replication projects. Journal of the Royal Statistical Society: Series A (Statistics in Society), 183(3), 1145–1166. 10.1111/rssa.12572

[bib26] Michell, J. (2003). Measurement: A beginner’s guide. Journal of Applied Measurement, 4(4), 298–308. PubMed: 14523251 14523251

[bib27] Papadimitropoulou, K., Stijnen, T., Dekkers, O. M., & Cessie, S. le. (2019). One-stage random effects meta-analysis using linear mixed models for aggregate continuous outcome data. Research Synthesis Methods, 10(3), 360–375. https://doi.org/10.1002/jrsm.1331, PubMed: 30523676 30523676 10.1002/jrsm.1331 PMC6767371

[bib28] R Core Team. (2021). R: A language and environment for statistical computing [Computer software]. R Foundation for Statistical Computing. https://www.R-project.org/

[bib29] Riley, R. D., Debray, T. P., Fisher, D., Hattle, M., Marlin, N., Hoogland, J., Gueyffier, F., Staessen, J. A., Wang, J., Moons, K. G. M., Reitsma, J. B., & Ensor, J. (2020). Individual participant data meta-analysis to examine interactions between treatment effect and participant-level covariates: Statistical recommendations for conduct and planning. Statistics in Medicine, 39(15), 2115–2137. https://doi.org/10.1002/sim.8516, PubMed: 32350891 32350891 10.1002/sim.8516 PMC7401032

[bib30] Rosenthal, R., Cooper, H., & Hedges, L. (1994). Parametric measures of effect size. The Handbook of Research Synthesis, 621(2), 231–244.

[bib31] Sung, Y. J., Schwander, K., Arnett, D. K., Kardia, S. L., Rankinen, T., Bouchard, C., Boerwinkle, E., Hunt, S. C., & Rao, D. C. C. (2014). An empirical comparison of meta-analysis and mega-analysis of individual participant data for identifying gene-environment interactions. Genetic Epidemiology, 38(4), 369–378. https://doi.org/10.1002/gepi.21800, PubMed: 24719363 24719363 10.1002/gepi.21800 PMC4332385

[bib32] van Aert, R. C. (2020). Analyzing data of a multi-lab replication project with individual participant data meta-analysis: A tutorial. MetaArXiv. https://osf.io/preprints/metaarxiv/9tmua/. 10.31222/osf.io/9tmua

[bib33] Verhage, M. L., Schuengel, C., Duschinsky, R., IJzendoorn, M. H. van, Fearon, R. P., Madigan, S., Roisman, G. I., Bakermans-Kranenburg, M. J., & Oosterman, M. (2020). The collaboration on attachment transmission synthesis (cats): A move to the level of individual-participant-data meta-analysis. Current Directions in Psychological Science, 29(2), 199–206. https://doi.org/10.1177/0963721420904967, PubMed: 32655212 32655212 10.1177/0963721420904967 PMC7324077

[bib34] Viechtbauer, W., & Viechtbauer, M. W. (2015). Package metafor. The Comprehensive R Archive Network. Package Metafor. CRAN. https://Cran.R-Project.Org/Web/Packages/Metafor/Metafor.Pdf

[bib35] Werker, J. F., & Tees, R. (1984). Cross-language speech perception. Infant Behavior and Development, 7, 49–63. 10.1016/S0163-6383(84)80022-3

[bib36] Zhang, Z. (2016). Multiple imputation with multivariate imputation by chained equation (mice) package. Annals of Translational Medicine, 4(2). 10.3978/j.issn.2305-5839.2015.12.63 PMC4731595 26889483

