# Supplementary material for: Toward Cumulative Cognitive Science: A Comparison of Meta-Analysis, Mega-Analysis, and Hybrid Approaches
Source: Open Mind (Camb). 2021 Nov 25;5:154–73. doi: 10.1162/opmi_a_00048 (PMC8746126; doi:10.1162/opmi_a_00048)
Supplement: Supplementary file 1 [file opmi-05-154-s001.pdf]

Supplementary Materials for ‘Towards cumulative cognitive science…’

Ezequiel Koile & Alejandrina Cristia

Contents

Appendix A: Full model results **1**
Experiment 1 . . . 1
Meta-analysis day 1 (REML) . . . 1
Meta-analysis day 1 using DerSimonian-Laird (DL) . . . 2
Meta-analysis day 1 using Robumeta . . . 2
Meta-analysis test-retest (REML) . . . 3
Meta-analysis test-retest using DerSimonian-Laird (DL) . . . 4
Meta-analysis test-retest using Robumeta . . . 4
Mega-analysis day 1 . . . 5
Mega-analysis test-retest . . . 6
Hybrid . . . 7
Experiment 2: Synthetic data . . . 7
Homogeneous . . . 7

Appendix B: Hybrid re-analyses with intermediate number of data points **34**
Results of hybrid analyses for day 1 d . . . 36
Results of hybrid analyses for test-retest r . . . 37

Appendix C: Varying number of participants per experiment N **38**

Appendix A: Full model results

Experiment 1

Meta-analysis day 1 (REML)

This is the full model print-out of the analyses presented in the main manuscript.

```
d_calc=effects$PQ1/effects$SD1
d_var_calc <- (1 / effects$N) + (d_calc ^ 2 / (2 * effects$N))

rma(yi=d_calc, vi=d_var_calc,
    ni=effects$N,slab=effects$Keyname,method="REML")->meta_d1
meta_d1
```

```
##
## Random-Effects Model (k = 13; tau^2 estimator: REML)
##
## tau^2 (estimated amount of total heterogeneity): 0.4426 (SE = 0.2079)
## tau (square root of estimated tau^2 value):      0.6653
## I^2 (total heterogeneity / total variability):   91.92%
```

```
## H^2 (total variability / sampling variability):  12.38
##
## Test for Heterogeneity:
## Q(df = 12) = 101.3387, p-val < .0001
##
## Model Results:
##
## estimate      se    zval    pval    ci.lb   ci.ub
##   0.3460  0.1987  1.7415  0.0816  -0.0434  0.7354  .
##
## ---
## Signif. codes:  0 '***' 0.001 '**' 0.01 '*' 0.05 '.' 0.1 ' ' 1
```

Meta-analysis day 1 using DerSimonian-Laird (DL)

We repeat the analyses using DerSimonian-Laird (DL) random effects model, instead of REML. DL is the parameter used in the original Cristia et al.'s study.

```
d_calc=effects$PQ1/effects$SD1
d_var_calc <- (1 / effects$N) + (d_calc ^ 2 / (2 * effects$N))

rma(yi=d_calc, vi=d_var_calc,
    ni=effects$N,slab=effects$Keyname,method="DL")->main_es
main_es
```

```
##
## Random-Effects Model (k = 13; tau^2 estimator: DL)
##
## tau^2 (estimated amount of total heterogeneity): 0.2896 (SE = 0.1613)
## tau (square root of estimated tau^2 value):      0.5382
## I^2 (total heterogeneity / total variability):   88.16%
## H^2 (total variability / sampling variability):  8.44
##
## Test for Heterogeneity:
## Q(df = 12) = 101.3387, p-val < .0001
##
## Model Results:
##
## estimate      se    zval    pval   ci.lb   ci.ub
##   0.3246  0.1655  1.9608  0.0499  0.0001  0.6490  *
##
## ---
## Signif. codes:  0 '***' 0.001 '**' 0.01 '*' 0.05 '.' 0.1 ' ' 1
```

Results do not change greatly our conclusions.

Meta-analysis day 1 using Robumeta

Here, we use robumeta instead of metafor. Robumeta is a package that handles better correlated structure. As discussed in the main paper, some infants were tested repeatedly (in LabC) and further each lab contributed data from multiple infant groups. Thus, there is some covariance structure that is not captured in the analyses above. We therefore repeat our analyses using a method that employs robust variance estimation to address this by considering the hierarchical data in this dataset.

```
effects$d_calc=effects$PQ1/effects$SD1
effects$d_var_calc <- (1 / effects$N) + (effects$d_calc ^ 2 / (2 * effects$N))
```

```r
effects$lab<-gsub(".*Lab","",effects$Keyname)

meta_d1 <- robu(d_calc ~1, data=effects,
                modelweights = "HIER", studynum = lab,
                var.eff.size = d_var_calc, small = T)
meta_d1
```

```
## RVE: Hierarchical Effects Model with Small-Sample Corrections
##
## Model: d_calc ~ 1
##
## Number of clusters = 4
## Number of outcomes = 13 (min = 1 , mean = 3.25 , median = 3.5 , max = 5 )
## Omega.sq = 0
## Tau.sq = 0.4112094
##
##                Estimate StdErr t-value  dfs P(|t|>) 95% CI.L 95% CI.U Sig
## 1 X.Intercept.    0.342  0.312     1.1 2.17   0.379   -0.903     1.59
## ---
## Signif. codes: < .01 *** < .05 ** < .10 *
## ---
## Note: If df < 4, do not trust the results
```

Here we do see quite different results, namely that the main effect for the first day of testing is not significant. This is due mainly to the fact that only 4 fully independent clusters are considered, with degrees of freedom thus greatly reduced, and the standard error for the intercept is greater than in the other analysis.

A sensitivity analysis could have been attempted if the datasets could have been studied with the correlated effects approach, but this dataset contains hierarchical structure, and thus a sensitivity analysis is not possible. Such an analysis would be recommended for meta-analyses in which multiple effect sizes are drawn from the same participants' data, a case in which a sensitivity analysis could reveal the consequences for overall conclusions of different levels of correlation among these effect sizes.

Meta-analysis test-retest (REML)

This is the full model print-out of the analyses presented in the main manuscript.

```r
rma(ri=effects$r, ni=effects$N,measure="ZCOR",slab=effects$Keyname,method="REML")->meta_r
meta_r
```

```
##
## Random-Effects Model (k = 13; tau^2 estimator: REML)
##
## tau^2 (estimated amount of total heterogeneity): 0.0638 (SE = 0.0444)
## tau (square root of estimated tau^2 value):      0.2526
## I^2 (total heterogeneity / total variability):   63.33%
## H^2 (total variability / sampling variability):  2.73
##
## Test for Heterogeneity:
## Q(df = 12) = 33.9942, p-val = 0.0007
##
## Model Results:
##
## estimate      se    zval    pval    ci.lb   ci.ub
##   0.0636  0.0931  0.6833  0.4944  -0.1189  0.2462
##
```

```
## ---
## Signif. codes:  0 '***' 0.001 '**' 0.01 '*' 0.05 '.' 0.1 ' ' 1
```

Meta-analysis test-retest using DerSimonian-Laird (DL)

We re-do the meta-analysis for test-retest using DL, the parameter used in Cristia et al's study. Conclusions do not change.

```r
rma(ri=effects$r, ni=effects$N,measure="ZCOR",slab=effects$Keyname,method="DL")->main_trt
main_trt
```

```
##
## Random-Effects Model (k = 13; tau^2 estimator: DL)
##
## tau^2 (estimated amount of total heterogeneity): 0.0677 (SE = 0.0472)
## tau (square root of estimated tau^2 value):      0.2603
## I^2 (total heterogeneity / total variability):   64.70%
## H^2 (total variability / sampling variability):  2.83
##
## Test for Heterogeneity:
## Q(df = 12) = 33.9942, p-val = 0.0007
##
## Model Results:
##
## estimate      se    zval    pval    ci.lb   ci.ub
##   0.0643  0.0949  0.6772  0.4983  -0.1217  0.2503
##
## ---
## Signif. codes:  0 '***' 0.001 '**' 0.01 '*' 0.05 '.' 0.1 ' ' 1
```

Meta-analysis test-retest using Robumeta

We re-do the meta-analysis for test-retest using robumeta. Conclusions do not change.

```r
effects$r_var <-  (1- effects$r^2)^2/(effects$N-1)

meta_r <- robu(r ~1, data=effects, modelweights = "HIER",
               studynum = lab, var.eff.size = r_var, small = T)
meta_r
```

```
## RVE: Hierarchical Effects Model with Small-Sample Corrections
##
## Model: r ~ 1
##
## Number of clusters = 4
## Number of outcomes = 13 (min = 1 , mean = 3.25 , median = 3.5 , max = 5 )
## Omega.sq = 0.1072692
## Tau.sq = 0
##
##                Estimate StdErr t-value  dfs P(|t|>) 95% CI.L 95% CI.U Sig
## 1 X.Intercept.   0.0792 0.0833   0.952 2.36   0.428   -0.231    0.389
## ---
## Signif. codes: < .01 *** < .05 ** < .10 *
## ---
## Note: If df < 4, do not trust the results
```

Mega-analysis day 1

```
#rearrange dataset to make paired observations also contain data about study-level results
pairs2 <- merge(pairs, PQs, "study", sort = TRUE)

colnames(pairs2)[colnames(pairs2)=="pq_day1"] <-  "pq1"
colnames(pairs2)[colnames(pairs2)=="pq_day2"] <-  "pq2"
colnames(pairs2)[colnames(pairs2)=="mean_pq1"] <-  "mean1"
colnames(pairs2)[colnames(pairs2)=="SD_pq1"] <-  "sd1"
colnames(pairs2)[colnames(pairs2)=="mean_pq2"] <-  "mean2"
colnames(pairs2)[colnames(pairs2)=="SD_pq2"] <-  "sd2"
colnames(pairs2)[colnames(pairs2)=="SE_pq1"] <-  "se1"
colnames(pairs2)[colnames(pairs2)=="SE_pq2"] <-  "se2"

#d1 and d2 are initially group-level
#replace with child-level d for day 1 as the child's PQ1 divided by the SD of the group
pairs2$d1 <- pairs2$pq1/pairs2$sd1
pairs2$d2 <- pairs2$pq2/pairs2$sd2

#adding kid as random effect (only described in SM):
#md1ks: Mega-analysis for d1, with Kid and Study as random effects
pairs2$kid <- as.factor(pairs2$kid)
md1ks = lmer(d1 ~ 1 + (1 | kid) + (1 | study),data=pairs2)

#kid is negligible for d1, we can only consider study:
#mega_d1: Mega-analysis for d1, with Study as a random effect
```

With random intercept for children and study

```
mega_d1 = lmer(d1 ~ 1 + (1 | study),data=pairs2)

summary(mega_d1)
```

With random intercept for study only

```
## Linear mixed model fit by REML. t-tests use Satterthwaite's method [
## lmerModLmerTest]
## Formula: d1 ~ 1 + (1 | study)
##    Data: pairs2
##
## REML criterion at convergence: 1199
##
## Scaled residuals:
##      Min       1Q   Median       3Q      Max
## -3.10123 -0.62140 -0.09487  0.67613  2.65111
##
## Random effects:
##  Groups   Name        Variance Std.Dev.
##  study    (Intercept) 0.6863   0.8284
##  Residual             1.0010   1.0005
## Number of obs: 409, groups:  study, 13
##
## Fixed effects:
```

```
##              Estimate Std. Error      df t value Pr(>|t|)
## (Intercept)   0.4206     0.2377 11.1874   1.769    0.104
```

Mega-analysis test-retest

```r
#adding kid as random effect (only described in SM), we calculate the correlation:
#mrks: Model for r, with Kid and Study as random effects
mrks =lmer(pq_day2 ~ pq_day1 + (1 | kid) + (1 + pq_day1 | study),data=pairs,
           control=lmerControl(optimizer="bobyqa", optCtrl=list(maxfun=2e5)))

summary(mrks)
```

With random intercept for child and study

```
## Linear mixed model fit by REML. t-tests use Satterthwaite's method [
## lmerModLmerTest]
## Formula: pq_day2 ~ pq_day1 + (1 | kid) + (1 + pq_day1 | study)
##    Data: pairs
## Control: lmerControl(optimizer = "bobyqa", optCtrl = list(maxfun = 2e+05))
##
## REML criterion at convergence: -105
##
## Scaled residuals:
##     Min      1Q  Median      3Q     Max
## -3.0863 -0.5696  0.0184  0.5911  3.4753
##
## Random effects:
##  Groups   Name        Variance Std.Dev. Corr
##  kid      (Intercept) 0.002705 0.05201
##  study    (Intercept) 0.019194 0.13854
##           pq_day1     0.041221 0.20303  0.64
##  Residual             0.037581 0.19386
## Number of obs: 409, groups:  kid, 300; study, 13
##
## Fixed effects:
##              Estimate Std. Error       df t value Pr(>|t|)
## (Intercept)  0.06694    0.04071 11.47099   1.644    0.127
## pq_day1      0.09818    0.07629 10.94434   1.287    0.225
##
## Correlation of Fixed Effects:
##         (Intr)
## pq_day1 0.387
```

```r
#kid is negligible for r, we can only consider study:
#mega_r: Model for r, with Study as a random effect


mega_r =lmer(pq_day2 ~ pq_day1 + (1 + pq_day1 | study),data=pairs,
             control=lmerControl(optimizer="bobyqa", optCtrl=list(maxfun=2e5)))
summary(mega_r)
```

With random intercept for study only

```
## Linear mixed model fit by REML. t-tests use Satterthwaite's method [
## lmerModLmerTest]
```

```
## Formula: pq_day2 ~ pq_day1 + (1 + pq_day1 | study)
##    Data: pairs
## Control: lmerControl(optimizer = "bobyqa", optCtrl = list(maxfun = 2e+05))
##
## REML criterion at convergence: -104.7
##
## Scaled residuals:
##     Min      1Q  Median      3Q     Max
## -3.1278 -0.5912  0.0074  0.5808  3.6056
##
## Random effects:
##  Groups   Name        Variance Std.Dev. Corr
##  study    (Intercept) 0.01932  0.1390
##           pq_day1     0.04295  0.2072   0.65
##  Residual             0.04024  0.2006
## Number of obs: 409, groups:  study, 13
##
## Fixed effects:
##             Estimate Std. Error       df t value Pr(>|t|)
## (Intercept)  0.06632    0.04078 11.48277   1.626    0.131
## pq_day1      0.09513    0.07723 11.09731   1.232    0.244
##
## Correlation of Fixed Effects:
##         (Intr)
## pq_day1 0.402
```

Hybrid

There are 100 x 2 models fit, which seems too many to show. Please see main manuscript for code, if you feel like it would be useful to print out their summaries.

Experiment 2: Synthetic data

Homogeneous

```r
effects$d_calc=effects$group_PQ1/effects$group_SD1
effects$d_var_calc <- (1 / effects$N) + (effects$d_calc ^ 2 / (2 * effects$N))

allds=allrs=NULL
for(thisS in myS) for(thisR in myR){

  print(paste("**Output for",thisS,"effect size on day 1, and", thisR,
              "test-retest correlation (homog., meta)**"))
  print(paste("*Meta-analysis: day 1 d*"))
  rma(yi=effects$d_calc, vi=effects$d_var_calc,
      ni=effects$N,slab=effects$Keyname,method="REML",
      subset=c(effects$day1size==thisS & effects$r==thisR))->meta_d1

  print(meta_d1)

  allds=rbind(allds,cbind(thisS,thisR,meta_d1$b,meta_d1$se))

    print(paste("*Meta-analysis: test-retest r*"))
```

```
    rma(ri=effects$r, ni=effects$N,measure="ZCOR",slab=effects$Keyname,
        method="REML",subset=c(effects$day1size==thisS & effects$r==thisR))->meta_r

    print(meta_r)

  allrs=rbind(allrs,
              cbind(thisS,thisR,meta_r$b,meta_r$se, tanh(meta_r$b),
                    (1-tanh(meta_r$b)^2)*meta_r$se))
}
```

Meta-analyses

```
## [1] "**Output for 0 effect size on day 1, and 0.15 test-retest correlation (homog., meta)**"
## [1] "*Meta-analysis: day 1 d*"
##
## Random-Effects Model (k = 10; tau^2 estimator: REML)
##
## tau^2 (estimated amount of total heterogeneity): 0.6098 (SE = 0.2935)
## tau (square root of estimated tau^2 value):      0.7809
## I^2 (total heterogeneity / total variability):   98.06%
## H^2 (total variability / sampling variability):  51.54
##
## Test for Heterogeneity:
## Q(df = 9) = 329.7555, p-val < .0001
##
## Model Results:
##
## estimate      se    zval    pval    ci.lb   ci.ub
##   0.0204  0.2495  0.0819  0.9347  -0.4686  0.5095
##
## ---
## Signif. codes:  0 '***' 0.001 '**' 0.01 '*' 0.05 '.' 0.1 ' ' 1
##
## [1] "*Meta-analysis: test-retest r*"
##
## Random-Effects Model (k = 10; tau^2 estimator: REML)
##
## tau^2 (estimated amount of total heterogeneity): 0 (SE = 0.0049)
## tau (square root of estimated tau^2 value):      0
## I^2 (total heterogeneity / total variability):   0.00%
## H^2 (total variability / sampling variability):  1.00
##
## Test for Heterogeneity:
## Q(df = 9) = 0.0000, p-val = 1.0000
##
## Model Results:
##
## estimate      se    zval    pval   ci.lb   ci.ub
##   0.1511  0.0321  4.7072  <.0001  0.0882  0.2141  ***
##
## ---
## Signif. codes:  0 '***' 0.001 '**' 0.01 '*' 0.05 '.' 0.1 ' ' 1
##
## [1] "**Output for 0 effect size on day 1, and 0.5 test-retest correlation (homog., meta)**"
```

```
## [1] "*Meta-analysis: day 1 d*"
##
## Random-Effects Model (k = 10; tau^2 estimator: REML)
##
## tau^2 (estimated amount of total heterogeneity): 0.7636 (SE = 0.3663)
## tau (square root of estimated tau^2 value):      0.8738
## I^2 (total heterogeneity / total variability):   98.40%
## H^2 (total variability / sampling variability):  62.53
##
## Test for Heterogeneity:
## Q(df = 9) = 376.5380, p-val < .0001
##
## Model Results:
##
## estimate      se     zval    pval    ci.lb   ci.ub
##  -0.0338  0.2788  -0.1214  0.9034  -0.5802  0.5125
##
## ---
## Signif. codes:  0 '***' 0.001 '**' 0.01 '*' 0.05 '.' 0.1 ' ' 1
##
## [1] "*Meta-analysis: test-retest r*"
##
## Random-Effects Model (k = 10; tau^2 estimator: REML)
##
## tau^2 (estimated amount of total heterogeneity): 0 (SE = 0.0049)
## tau (square root of estimated tau^2 value):      0
## I^2 (total heterogeneity / total variability):   0.00%
## H^2 (total variability / sampling variability):  1.00
##
## Test for Heterogeneity:
## Q(df = 9) = 0.0000, p-val = 1.0000
##
## Model Results:
##
## estimate      se     zval    pval   ci.lb   ci.ub
##   0.5493  0.0321  17.1080  <.0001  0.4864  0.6122  ***
##
## ---
## Signif. codes:  0 '***' 0.001 '**' 0.01 '*' 0.05 '.' 0.1 ' ' 1
##
## [1] "**Output for 0 effect size on day 1, and 0.85 test-retest correlation (homog., meta)**"
## [1] "*Meta-analysis: day 1 d*"
##
## Random-Effects Model (k = 10; tau^2 estimator: REML)
##
## tau^2 (estimated amount of total heterogeneity): 0.7315 (SE = 0.3512)
## tau (square root of estimated tau^2 value):      0.8553
## I^2 (total heterogeneity / total variability):   98.34%
## H^2 (total variability / sampling variability):  60.40
##
## Test for Heterogeneity:
## Q(df = 9) = 357.3882, p-val < .0001
##
## Model Results:
```

```
##
## estimate      se    zval    pval    ci.lb   ci.ub
##   0.0312  0.2729  0.1144  0.9089  -0.5037  0.5662
##
## ---
## Signif. codes:  0 '***' 0.001 '**' 0.01 '*' 0.05 '.' 0.1 ' ' 1
##
## [1] "*Meta-analysis: test-retest r*"
##
## Random-Effects Model (k = 10; tau^2 estimator: REML)
##
## tau^2 (estimated amount of total heterogeneity): 0 (SE = 0.0049)
## tau (square root of estimated tau^2 value):      0
## I^2 (total heterogeneity / total variability):   0.00%
## H^2 (total variability / sampling variability):  1.00
##
## Test for Heterogeneity:
## Q(df = 9) = 0.0000, p-val = 1.0000
##
## Model Results:
##
## estimate      se     zval    pval   ci.lb   ci.ub
##   1.2562  0.0321  39.1227  <.0001  1.1932  1.3191  ***
##
## ---
## Signif. codes:  0 '***' 0.001 '**' 0.01 '*' 0.05 '.' 0.1 ' ' 1
##
## [1] "**Output for 0.3 effect size on day 1, and 0.15 test-retest correlation (homog., meta)**"
## [1] "*Meta-analysis: day 1 d*"
##
## Random-Effects Model (k = 10; tau^2 estimator: REML)
##
## tau^2 (estimated amount of total heterogeneity): 0.6903 (SE = 0.3413)
## tau (square root of estimated tau^2 value):      0.8308
## I^2 (total heterogeneity / total variability):   96.11%
## H^2 (total variability / sampling variability):  25.68
##
## Test for Heterogeneity:
## Q(df = 9) = 339.1846, p-val < .0001
##
## Model Results:
##
## estimate      se    zval    pval   ci.lb   ci.ub
##   2.0308  0.2691  7.5470  <.0001  1.5034  2.5582  ***
##
## ---
## Signif. codes:  0 '***' 0.001 '**' 0.01 '*' 0.05 '.' 0.1 ' ' 1
##
## [1] "*Meta-analysis: test-retest r*"
##
## Random-Effects Model (k = 10; tau^2 estimator: REML)
##
## tau^2 (estimated amount of total heterogeneity): 0 (SE = 0.0049)
## tau (square root of estimated tau^2 value):      0
```

```
## I^2 (total heterogeneity / total variability):   0.00%
## H^2 (total variability / sampling variability):  1.00
##
## Test for Heterogeneity:
## Q(df = 9) = 0.0000, p-val = 1.0000
##
## Model Results:
##
## estimate      se    zval    pval   ci.lb   ci.ub
##   0.1511  0.0321  4.7072  <.0001  0.0882  0.2141  ***
##
## ---
## Signif. codes:  0 '***' 0.001 '**' 0.01 '*' 0.05 '.' 0.1 ' ' 1
##
## [1] "**Output for 0.3 effect size on day 1, and 0.5 test-retest correlation (homog., meta)**"
## [1] "*Meta-analysis: day 1 d*"
##
## Random-Effects Model (k = 10; tau^2 estimator: REML)
##
## tau^2 (estimated amount of total heterogeneity): 0.6358 (SE = 0.3151)
## tau (square root of estimated tau^2 value):      0.7973
## I^2 (total heterogeneity / total variability):   95.87%
## H^2 (total variability / sampling variability):  24.23
##
## Test for Heterogeneity:
## Q(df = 9) = 311.1326, p-val < .0001
##
## Model Results:
##
## estimate      se    zval    pval   ci.lb   ci.ub
##   1.9834  0.2585  7.6716  <.0001  1.4767  2.4901  ***
##
## ---
## Signif. codes:  0 '***' 0.001 '**' 0.01 '*' 0.05 '.' 0.1 ' ' 1
##
## [1] "*Meta-analysis: test-retest r*"
##
## Random-Effects Model (k = 10; tau^2 estimator: REML)
##
## tau^2 (estimated amount of total heterogeneity): 0 (SE = 0.0049)
## tau (square root of estimated tau^2 value):      0
## I^2 (total heterogeneity / total variability):   0.00%
## H^2 (total variability / sampling variability):  1.00
##
## Test for Heterogeneity:
## Q(df = 9) = 0.0000, p-val = 1.0000
##
## Model Results:
##
## estimate      se     zval    pval   ci.lb   ci.ub
##   0.5493  0.0321  17.1080  <.0001  0.4864  0.6122  ***
##
## ---
## Signif. codes:  0 '***' 0.001 '**' 0.01 '*' 0.05 '.' 0.1 ' ' 1
```

```
##
## [1] "**Output for 0.3 effect size on day 1, and 0.85 test-retest correlation (homog., meta)**"
## [1] "*Meta-analysis: day 1 d*"
##
## Random-Effects Model (k = 10; tau^2 estimator: REML)
##
## tau^2 (estimated amount of total heterogeneity): 0.8546 (SE = 0.4187)
## tau (square root of estimated tau^2 value):      0.9244
## I^2 (total heterogeneity / total variability):   96.94%
## H^2 (total variability / sampling variability):  32.72
##
## Test for Heterogeneity:
## Q(df = 9) = 379.0271, p-val < .0001
##
## Model Results:
##
## estimate      se    zval    pval   ci.lb   ci.ub
##   1.9893  0.2980  6.6744  <.0001  1.4051  2.5734  ***
##
## ---
## Signif. codes:  0 '***' 0.001 '**' 0.01 '*' 0.05 '.' 0.1 ' ' 1
##
## [1] "*Meta-analysis: test-retest r*"
##
## Random-Effects Model (k = 10; tau^2 estimator: REML)
##
## tau^2 (estimated amount of total heterogeneity): 0 (SE = 0.0049)
## tau (square root of estimated tau^2 value):      0
## I^2 (total heterogeneity / total variability):   0.00%
## H^2 (total variability / sampling variability):  1.00
##
## Test for Heterogeneity:
## Q(df = 9) = 0.0000, p-val = 1.0000
##
## Model Results:
##
## estimate      se     zval    pval   ci.lb   ci.ub
##   1.2562  0.0321  39.1227  <.0001  1.1932  1.3191  ***
##
## ---
## Signif. codes:  0 '***' 0.001 '**' 0.01 '*' 0.05 '.' 0.1 ' ' 1
##
## [1] "**Output for 0.6 effect size on day 1, and 0.15 test-retest correlation (homog., meta)**"
## [1] "*Meta-analysis: day 1 d*"
##
## Random-Effects Model (k = 10; tau^2 estimator: REML)
##
## tau^2 (estimated amount of total heterogeneity): 0.7012 (SE = 0.3745)
## tau (square root of estimated tau^2 value):      0.8374
## I^2 (total heterogeneity / total variability):   89.33%
## H^2 (total variability / sampling variability):  9.37
##
## Test for Heterogeneity:
## Q(df = 9) = 90.2017, p-val < .0001
```

```
##
## Model Results:
##
## estimate      se     zval    pval   ci.lb   ci.ub
##   4.0015  0.2819  14.1932  <.0001  3.4489  4.5541  ***
##
## ---
## Signif. codes:  0 '***' 0.001 '**' 0.01 '*' 0.05 '.' 0.1 ' ' 1
##
## [1] "*Meta-analysis: test-retest r*"
##
## Random-Effects Model (k = 10; tau^2 estimator: REML)
##
## tau^2 (estimated amount of total heterogeneity): 0 (SE = 0.0049)
## tau (square root of estimated tau^2 value):      0
## I^2 (total heterogeneity / total variability):   0.00%
## H^2 (total variability / sampling variability):  1.00
##
## Test for Heterogeneity:
## Q(df = 9) = 0.0000, p-val = 1.0000
##
## Model Results:
##
## estimate      se    zval    pval   ci.lb   ci.ub
##   0.1511  0.0321  4.7072  <.0001  0.0882  0.2141  ***
##
## ---
## Signif. codes:  0 '***' 0.001 '**' 0.01 '*' 0.05 '.' 0.1 ' ' 1
##
## [1] "**Output for 0.6 effect size on day 1, and 0.5 test-retest correlation (homog., meta)**"
## [1] "*Meta-analysis: day 1 d*"
##
## Random-Effects Model (k = 10; tau^2 estimator: REML)
##
## tau^2 (estimated amount of total heterogeneity): 0.6988 (SE = 0.3740)
## tau (square root of estimated tau^2 value):      0.8360
## I^2 (total heterogeneity / total variability):   89.21%
## H^2 (total variability / sampling variability):  9.27
##
## Test for Heterogeneity:
## Q(df = 9) = 114.1250, p-val < .0001
##
## Model Results:
##
## estimate      se     zval    pval   ci.lb   ci.ub
##   4.0352  0.2817  14.3230  <.0001  3.4830  4.5874  ***
##
## ---
## Signif. codes:  0 '***' 0.001 '**' 0.01 '*' 0.05 '.' 0.1 ' ' 1
##
## [1] "*Meta-analysis: test-retest r*"
##
## Random-Effects Model (k = 10; tau^2 estimator: REML)
##
```

```
## tau^2 (estimated amount of total heterogeneity): 0 (SE = 0.0049)
## tau (square root of estimated tau^2 value):      0
## I^2 (total heterogeneity / total variability):   0.00%
## H^2 (total variability / sampling variability):  1.00
##
## Test for Heterogeneity:
## Q(df = 9) = 0.0000, p-val = 1.0000
##
## Model Results:
##
## estimate      se     zval    pval   ci.lb   ci.ub
##   0.5493  0.0321  17.1080  <.0001  0.4864  0.6122  ***
##
## ---
## Signif. codes:  0 '***' 0.001 '**' 0.01 '*' 0.05 '.' 0.1 ' ' 1
##
## [1] "**Output for 0.6 effect size on day 1, and 0.85 test-retest correlation (homog., meta)**"
## [1] "*Meta-analysis: day 1 d*"
##
## Random-Effects Model (k = 10; tau^2 estimator: REML)
##
## tau^2 (estimated amount of total heterogeneity): 0.6162 (SE = 0.3346)
## tau (square root of estimated tau^2 value):      0.7850
## I^2 (total heterogeneity / total variability):   87.92%
## H^2 (total variability / sampling variability):  8.28
##
## Test for Heterogeneity:
## Q(df = 9) = 108.0341, p-val < .0001
##
## Model Results:
##
## estimate      se     zval    pval   ci.lb   ci.ub
##   4.0217  0.2665  15.0921  <.0001  3.4994  4.5440  ***
##
## ---
## Signif. codes:  0 '***' 0.001 '**' 0.01 '*' 0.05 '.' 0.1 ' ' 1
##
## [1] "*Meta-analysis: test-retest r*"
##
## Random-Effects Model (k = 10; tau^2 estimator: REML)
##
## tau^2 (estimated amount of total heterogeneity): 0 (SE = 0.0049)
## tau (square root of estimated tau^2 value):      0
## I^2 (total heterogeneity / total variability):   0.00%
## H^2 (total variability / sampling variability):  1.00
##
## Test for Heterogeneity:
## Q(df = 9) = 0.0000, p-val = 1.0000
##
## Model Results:
##
## estimate      se     zval    pval   ci.lb   ci.ub
##   1.2562  0.0321  39.1227  <.0001  1.1932  1.3191  ***
##
```

```
## ---
## Signif. codes:  0 '***' 0.001 '**' 0.01 '*' 0.05 '.' 0.1 ' ' 1
```

```
  colnames(allds) <- c("day1size","sim_r","d","SE(d)")
  colnames(allrs) <- c("day1size","sim_r","rz","SE(rz)","r","SE(r)")

  allds_meta_hom <- allds
  allrs_meta_hom <- allrs
```

```
# generate pq1d at the child level dividing by group-based sd
dt$Keyname=paste("day1size",dt$day1size,"r",dt$r,"study",dt$study)
dt1=merge(effects[,c("group_PQ1","group_SD1","r_calc",
                     "N","Keyname","d_calc","d_var_calc")],dt, by="Keyname")
dt1$d1=dt1$PQ1/dt1$group_SD1

allb_d1s=allb_rs=NULL
for(thisS in myS) for(thisR in myR){

  #print(c(thisS,thisR))
  print(paste("**Output for",thisS,"effect size on day 1, and",
              thisR, "test-retest correlation (homog., mega)**"))

  print(paste("*Mega-analysis: day 1 d*"))
  mega_d1 = lmer(d1 ~ 1 + (1 | study),data=dt1,subset=c(day1size==thisS & r==thisR))
  print(mega_d1)

  ran.variance.d <- as.data.frame(VarCorr(mega_d1))
  allb_d1s=rbind(allb_d1s,
              cbind(thisS, thisR, summary(mega_d1)$coefficients["(Intercept)","Estimate"],
                    summary(mega_d1)$coefficients["(Intercept)","Std. Error"],
                    100*round(ran.variance.d$vcov[1]/
                                (ran.variance.d$vcov[1]+ran.variance.d$vcov[2]),2)))

  print(paste("*Mega-analysis: test-retest r*"))
  mega_r =lmer(PQ2 ~ PQ1 + (1 + PQ1 | study),data=dt1,
subset=c(day1size==thisS & r==thisR),control=lmerControl(optimizer="bobyqa"))
  print(mega_r)

 ran.variance.r <- as.data.frame(VarCorr(mega_r))
  allb_rs=rbind(allb_rs,
              cbind(thisS, thisR,
                    summary(mega_r)$coefficients["(Intercept)","Estimate"],
                    summary(mega_r)$coefficients["PQ1","Estimate"],
                    summary(mega_r)$coefficients["(Intercept)","Std. Error"],
                    summary(mega_r)$coefficients["PQ1","Std. Error"],
                    100*round(ran.variance.r$vcov[1]/(ran.variance.r$vcov[1]+
ran.variance.r$vcov[2]+ran.variance.r$vcov[4]),2),
                    100*round(ran.variance.r$vcov[2]/
 (ran.variance.r$vcov[1]+ran.variance.r$vcov[2]+ran.variance.r$vcov[4]),2)))


}
```

Mega-analyses

```
## [1] "**Output for 0 effect size on day 1, and 0.15 test-retest correlation (homog., mega)**"
## [1] "*Mega-analysis: day 1 d*"
## Linear mixed model fit by REML ['lmerModLmerTest']
## Formula: d1 ~ 1 + (1 | study)
##    Data: dt1
##  Subset: c(day1size == thisS & r == thisR)
## REML criterion at convergence: 2879.302
## Random effects:
##  Groups   Name        Std.Dev.
##  study    (Intercept) 0.7904
##  Residual             1.0000
## Number of obs: 1000, groups:  study, 10
## Fixed Effects:
## (Intercept)
##     0.01801
## [1] "*Mega-analysis: test-retest r*"
## Linear mixed model fit by REML ['lmerModLmerTest']
## Formula: PQ2 ~ PQ1 + (1 + PQ1 | study)
##    Data: dt1
##  Subset: c(day1size == thisS & r == thisR)
## REML criterion at convergence: -1009.855
## Random effects:
##  Groups   Name        Std.Dev. Corr
##  study    (Intercept) 0.1147
##           PQ1         0.1940   -0.06
##  Residual             0.1414
## Number of obs: 1000, groups:  study, 10
## Fixed Effects:
## (Intercept)          PQ1
##   -0.008502     0.160195
## [1] "**Output for 0 effect size on day 1, and 0.5 test-retest correlation (homog., mega)**"
## [1] "*Mega-analysis: day 1 d*"
## Linear mixed model fit by REML ['lmerModLmerTest']
## Formula: d1 ~ 1 + (1 | study)
##    Data: dt1
##  Subset: c(day1size == thisS & r == thisR)
## REML criterion at convergence: 2881.317
## Random effects:
##  Groups   Name        Std.Dev.
##  study    (Intercept) 0.8854
##  Residual             1.0000
## Number of obs: 1000, groups:  study, 10
## Fixed Effects:
## (Intercept)
##    -0.03765
## [1] "*Mega-analysis: test-retest r*"
## Linear mixed model fit by REML ['lmerModLmerTest']
## Formula: PQ2 ~ PQ1 + (1 + PQ1 | study)
##    Data: dt1
##  Subset: c(day1size == thisS & r == thisR)
## REML criterion at convergence: -1214.989
## Random effects:
```

```
##  Groups   Name        Std.Dev. Corr
##  study    (Intercept) 0.0632
##           PQ1         0.1860   -0.06
##  Residual             0.1281
## Number of obs: 1000, groups:  study, 10
## Fixed Effects:
## (Intercept)          PQ1
##    0.006315     0.512938
## [1] "**Output for 0 effect size on day 1, and 0.85 test-retest correlation (homog., mega)**"
## [1] "*Mega-analysis: day 1 d*"
## Linear mixed model fit by REML ['lmerModLmerTest']
## Formula: d1 ~ 1 + (1 | study)
##    Data: dt1
##  Subset: c(day1size == thisS & r == thisR)
## REML criterion at convergence: 2880.948
## Random effects:
##  Groups   Name        Std.Dev.
##  study    (Intercept) 0.8672
##  Residual             1.0000
## Number of obs: 1000, groups:  study, 10
## Fixed Effects:
## (Intercept)
##     0.02762
## [1] "*Mega-analysis: test-retest r*"
## Linear mixed model fit by REML ['lmerModLmerTest']
## Formula: PQ2 ~ PQ1 + (1 + PQ1 | study)
##    Data: dt1
##  Subset: c(day1size == thisS & r == thisR)
## REML criterion at convergence: -2217.862
## Random effects:
##  Groups   Name        Std.Dev. Corr
##  study    (Intercept) 0.02537
##           PQ1         0.10742  -0.18
##  Residual             0.07780
## Number of obs: 1000, groups:  study, 10
## Fixed Effects:
## (Intercept)          PQ1
##     0.00725      0.83623
## [1] "**Output for 0.3 effect size on day 1, and 0.15 test-retest correlation (homog., mega)**"
## [1] "*Mega-analysis: day 1 d*"
## Linear mixed model fit by REML ['lmerModLmerTest']
## Formula: d1 ~ 1 + (1 | study)
##    Data: dt1
##  Subset: c(day1size == thisS & r == thisR)
## REML criterion at convergence: 2880.247
## Random effects:
##  Groups   Name        Std.Dev.
##  study    (Intercept) 0.8336
##  Residual             1.0000
## Number of obs: 1000, groups:  study, 10
## Fixed Effects:
## (Intercept)
##       2.045
## [1] "*Mega-analysis: test-retest r*"
```

```
## Linear mixed model fit by REML ['lmerModLmerTest']
## Formula: PQ2 ~ PQ1 + (1 + PQ1 | study)
##    Data: dt1
##  Subset: c(day1size == thisS & r == thisR)
## REML criterion at convergence: -989.5069
## Random effects:
##  Groups   Name        Std.Dev. Corr
##  study    (Intercept) 0.1344
##           PQ1         0.2170   -0.60
##  Residual             0.1429
## Number of obs: 1000, groups:  study, 10
## Fixed Effects:
## (Intercept)          PQ1
##      0.2560       0.1696
## [1] "**Output for 0.3 effect size on day 1, and 0.5 test-retest correlation (homog., mega)**"
## [1] "*Mega-analysis: day 1 d*"
## Linear mixed model fit by REML ['lmerModLmerTest']
## Formula: d1 ~ 1 + (1 | study)
##    Data: dt1
##  Subset: c(day1size == thisS & r == thisR)
## REML criterion at convergence: 2879.569
## Random effects:
##  Groups   Name        Std.Dev.
##  study    (Intercept) 0.8024
##  Residual             1.0000
## Number of obs: 1000, groups:  study, 10
## Fixed Effects:
## (Intercept)
##       1.998
## [1] "*Mega-analysis: test-retest r*"
## Linear mixed model fit by REML ['lmerModLmerTest']
## Formula: PQ2 ~ PQ1 + (1 + PQ1 | study)
##    Data: dt1
##  Subset: c(day1size == thisS & r == thisR)
## REML criterion at convergence: -1187.572
## Random effects:
##  Groups   Name        Std.Dev. Corr
##  study    (Intercept) 0.08935
##           PQ1         0.18368  -0.52
##  Residual             0.12964
## Number of obs: 1000, groups:  study, 10
## Fixed Effects:
## (Intercept)          PQ1
##      0.1474       0.4953
## [1] "**Output for 0.3 effect size on day 1, and 0.85 test-retest correlation (homog., mega)**"
## [1] "*Mega-analysis: day 1 d*"
## Linear mixed model fit by REML ['lmerModLmerTest']
## Formula: d1 ~ 1 + (1 | study)
##    Data: dt1
##  Subset: c(day1size == thisS & r == thisR)
## REML criterion at convergence: 2882.222
## Random effects:
##  Groups   Name        Std.Dev.
##  study    (Intercept) 0.9316
```

```
##  Residual             1.0000
## Number of obs: 1000, groups:  study, 10
## Fixed Effects:
## (Intercept)
##       2.004
## [1] "*Mega-analysis: test-retest r*"
## Linear mixed model fit by REML ['lmerModLmerTest']
## Formula: PQ2 ~ PQ1 + (1 + PQ1 | study)
##    Data: dt1
##  Subset: c(day1size == thisS & r == thisR)
## REML criterion at convergence: -2231.168
## Random effects:
##  Groups   Name        Std.Dev. Corr
##  study    (Intercept) 0.02953
##           PQ1         0.10498  -0.85
##  Residual             0.07753
## Number of obs: 1000, groups:  study, 10
## Fixed Effects:
## (Intercept)          PQ1
##     0.05401      0.82614
## [1] "**Output for 0.6 effect size on day 1, and 0.15 test-retest correlation (homog., mega)**"
## [1] "*Mega-analysis: day 1 d*"
## Linear mixed model fit by REML ['lmerModLmerTest']
## Formula: d1 ~ 1 + (1 | study)
##    Data: dt1
##  Subset: c(day1size == thisS & r == thisR)
## REML criterion at convergence: 2881.3
## Random effects:
##  Groups   Name        Std.Dev.
##  study    (Intercept) 0.8845
##  Residual             1.0000
## Number of obs: 1000, groups:  study, 10
## Fixed Effects:
## (Intercept)
##       4.037
## [1] "*Mega-analysis: test-retest r*"
## Linear mixed model fit by REML ['lmerModLmerTest']
## Formula: PQ2 ~ PQ1 + (1 + PQ1 | study)
##    Data: dt1
##  Subset: c(day1size == thisS & r == thisR)
## REML criterion at convergence: -964.8288
## Random effects:
##  Groups   Name        Std.Dev. Corr
##  study    (Intercept) 0.1794
##           PQ1         0.2097   -0.79
##  Residual             0.1446
## Number of obs: 1000, groups:  study, 10
## Fixed Effects:
## (Intercept)          PQ1
##      0.5106       0.1607
## [1] "**Output for 0.6 effect size on day 1, and 0.5 test-retest correlation (homog., mega)**"
## [1] "*Mega-analysis: day 1 d*"
## Linear mixed model fit by REML ['lmerModLmerTest']
## Formula: d1 ~ 1 + (1 | study)
```

```
##    Data: dt1
##  Subset: c(day1size == thisS & r == thisR)
## REML criterion at convergence: 2880.807
## Random effects:
##  Groups   Name        Std.Dev.
##  study    (Intercept) 0.8604
##  Residual             1.0000
## Number of obs: 1000, groups:  study, 10
## Fixed Effects:
## (Intercept)
##       4.067
## [1] "*Mega-analysis: test-retest r*"
## Linear mixed model fit by REML ['lmerModLmerTest']
## Formula: PQ2 ~ PQ1 + (1 + PQ1 | study)
##    Data: dt1
##  Subset: c(day1size == thisS & r == thisR)
## REML criterion at convergence: -1180.182
## Random effects:
##  Groups   Name        Std.Dev. Corr
##  study    (Intercept) 0.1367
##           PQ1         0.1887   -0.88
##  Residual             0.1303
## Number of obs: 1000, groups:  study, 10
## Fixed Effects:
## (Intercept)          PQ1
##      0.2963       0.5171
## [1] "**Output for 0.6 effect size on day 1, and 0.85 test-retest correlation (homog., mega)**"
## [1] "*Mega-analysis: day 1 d*"
## Linear mixed model fit by REML ['lmerModLmerTest']
## Formula: d1 ~ 1 + (1 | study)
##    Data: dt1
##  Subset: c(day1size == thisS & r == thisR)
## REML criterion at convergence: 2879.535
## Random effects:
##  Groups   Name        Std.Dev.
##  study    (Intercept) 0.8008
##  Residual             1.0000
## Number of obs: 1000, groups:  study, 10
## Fixed Effects:
## (Intercept)
##       4.052
## [1] "*Mega-analysis: test-retest r*"
## Linear mixed model fit by REML ['lmerModLmerTest']
## Formula: PQ2 ~ PQ1 + (1 + PQ1 | study)
##    Data: dt1
##  Subset: c(day1size == thisS & r == thisR)
## REML criterion at convergence: -2106.779
## Random effects:
##  Groups   Name        Std.Dev. Corr
##  study    (Intercept) 0.03122
##           PQ1         0.05335  -0.75
##  Residual             0.08276
## Number of obs: 1000, groups:  study, 10
## Fixed Effects:
```

```
## (Intercept)          PQ1
##     0.08225      0.86373
```

```r
colnames(allb_d1s)<-c("day1size","r","b0","SE(b0)","REvarexplained")

colnames(allb_rs)<-c("day1size","r","b0","b1","SE(b0)","SE(b1)",
                     "REvarexplained_int","REvarexplained_slope")

  allds_mega_hom <- allb_d1s
  allrs_mega_hom <- allb_rs
```

```r
effects$d_calc=effects$group_PQ1/effects$group_SD1
effects$d_var_calc <- (1 / effects$N) + (effects$d_calc ^ 2 / (2 * effects$N))

allds=allrs=NULL
for(thisS in myS) for(thisR in myR){
  print(paste("**Output for",thisS,"effect size on day 1, and", thisR,
             "test-retest correlation (heterog., meta)**"))

  print(paste("*Meta-analysis: day 1 d*"))
  rma(yi=effects$d_calc, vi=effects$d_var_calc, ni=effects$N,slab=effects$Keyname,method="REML",subset=

  print(meta_d1)

  allds=rbind(allds,cbind(thisS,thisR,meta_d1$b,meta_d1$se))

    print(paste("*Meta-analysis: test-retest r*"))

  rma(ri=effects$r, ni=effects$N,measure="ZCOR",
      slab=effects$Keyname,method="REML",
      subset=c(effects$day1size==thisS & effects$r==thisR))->meta_r
  print(meta_r)

  allrs=rbind(allrs,cbind(thisS,thisR,meta_r$b,meta_r$se,
                          tanh(meta_r$b), (1-tanh(meta_r$b)^2)*meta_r$se))

}
```

Meta-analyses

```
## [1] "**Output for 0 effect size on day 1, and 0.15 test-retest correlation (heterog., meta)**"
## [1] "*Meta-analysis: day 1 d*"
##
## Random-Effects Model (k = 10; tau^2 estimator: REML)
##
## tau^2 (estimated amount of total heterogeneity): 0.6098 (SE = 0.2935)
## tau (square root of estimated tau^2 value):      0.7809
## I^2 (total heterogeneity / total variability):   98.06%
## H^2 (total variability / sampling variability):  51.54
##
## Test for Heterogeneity:
## Q(df = 9) = 329.7555, p-val < .0001
##
## Model Results:
```

```
##
## estimate      se    zval    pval    ci.lb   ci.ub
##   0.0204  0.2495  0.0819  0.9347  -0.4686  0.5095
##
## ---
## Signif. codes:  0 '***' 0.001 '**' 0.01 '*' 0.05 '.' 0.1 ' ' 1
##
## [1] "*Meta-analysis: test-retest r*"
##
## Random-Effects Model (k = 10; tau^2 estimator: REML)
##
## tau^2 (estimated amount of total heterogeneity): 0 (SE = 0.0049)
## tau (square root of estimated tau^2 value):      0
## I^2 (total heterogeneity / total variability):   0.00%
## H^2 (total variability / sampling variability):  1.00
##
## Test for Heterogeneity:
## Q(df = 9) = 0.0000, p-val = 1.0000
##
## Model Results:
##
## estimate      se    zval    pval   ci.lb   ci.ub
##   0.1511  0.0321  4.7072  <.0001  0.0882  0.2141  ***
##
## ---
## Signif. codes:  0 '***' 0.001 '**' 0.01 '*' 0.05 '.' 0.1 ' ' 1
##
## [1] "**Output for 0 effect size on day 1, and 0.5 test-retest correlation (heterog., meta)**"
## [1] "*Meta-analysis: day 1 d*"
##
## Random-Effects Model (k = 10; tau^2 estimator: REML)
##
## tau^2 (estimated amount of total heterogeneity): 0.7636 (SE = 0.3663)
## tau (square root of estimated tau^2 value):      0.8738
## I^2 (total heterogeneity / total variability):   98.40%
## H^2 (total variability / sampling variability):  62.53
##
## Test for Heterogeneity:
## Q(df = 9) = 376.5380, p-val < .0001
##
## Model Results:
##
## estimate      se     zval    pval    ci.lb   ci.ub
##  -0.0338  0.2788  -0.1214  0.9034  -0.5802  0.5125
##
## ---
## Signif. codes:  0 '***' 0.001 '**' 0.01 '*' 0.05 '.' 0.1 ' ' 1
##
## [1] "*Meta-analysis: test-retest r*"
##
## Random-Effects Model (k = 10; tau^2 estimator: REML)
##
## tau^2 (estimated amount of total heterogeneity): 0 (SE = 0.0049)
## tau (square root of estimated tau^2 value):      0
```

```
## I^2 (total heterogeneity / total variability):   0.00%
## H^2 (total variability / sampling variability):  1.00
##
## Test for Heterogeneity:
## Q(df = 9) = 0.0000, p-val = 1.0000
##
## Model Results:
##
## estimate      se     zval    pval   ci.lb   ci.ub
##   0.5493  0.0321  17.1080  <.0001  0.4864  0.6122  ***
##
## ---
## Signif. codes:  0 '***' 0.001 '**' 0.01 '*' 0.05 '.' 0.1 ' ' 1
##
## [1] "**Output for 0 effect size on day 1, and 0.85 test-retest correlation (heterog., meta)**"
## [1] "*Meta-analysis: day 1 d*"
##
## Random-Effects Model (k = 10; tau^2 estimator: REML)
##
## tau^2 (estimated amount of total heterogeneity): 0.7315 (SE = 0.3512)
## tau (square root of estimated tau^2 value):      0.8553
## I^2 (total heterogeneity / total variability):   98.34%
## H^2 (total variability / sampling variability):  60.40
##
## Test for Heterogeneity:
## Q(df = 9) = 357.3882, p-val < .0001
##
## Model Results:
##
## estimate      se    zval    pval    ci.lb   ci.ub
##   0.0312  0.2729  0.1144  0.9089  -0.5037  0.5662
##
## ---
## Signif. codes:  0 '***' 0.001 '**' 0.01 '*' 0.05 '.' 0.1 ' ' 1
##
## [1] "*Meta-analysis: test-retest r*"
##
## Random-Effects Model (k = 10; tau^2 estimator: REML)
##
## tau^2 (estimated amount of total heterogeneity): 0 (SE = 0.0049)
## tau (square root of estimated tau^2 value):      0
## I^2 (total heterogeneity / total variability):   0.00%
## H^2 (total variability / sampling variability):  1.00
##
## Test for Heterogeneity:
## Q(df = 9) = 0.0000, p-val = 1.0000
##
## Model Results:
##
## estimate      se     zval    pval   ci.lb   ci.ub
##   1.2562  0.0321  39.1227  <.0001  1.1932  1.3191  ***
##
## ---
## Signif. codes:  0 '***' 0.001 '**' 0.01 '*' 0.05 '.' 0.1 ' ' 1
```

```
##
## [1] "**Output for 0.3 effect size on day 1, and 0.15 test-retest correlation (heterog., meta)**"
## [1] "*Meta-analysis: day 1 d*"
##
## Random-Effects Model (k = 10; tau^2 estimator: REML)
##
## tau^2 (estimated amount of total heterogeneity): 0.6903 (SE = 0.3413)
## tau (square root of estimated tau^2 value):      0.8308
## I^2 (total heterogeneity / total variability):   96.11%
## H^2 (total variability / sampling variability):  25.68
##
## Test for Heterogeneity:
## Q(df = 9) = 339.1846, p-val < .0001
##
## Model Results:
##
## estimate      se    zval    pval   ci.lb   ci.ub
##   2.0308  0.2691  7.5470  <.0001  1.5034  2.5582  ***
##
## ---
## Signif. codes:  0 '***' 0.001 '**' 0.01 '*' 0.05 '.' 0.1 ' ' 1
##
## [1] "*Meta-analysis: test-retest r*"
##
## Random-Effects Model (k = 10; tau^2 estimator: REML)
##
## tau^2 (estimated amount of total heterogeneity): 0 (SE = 0.0049)
## tau (square root of estimated tau^2 value):      0
## I^2 (total heterogeneity / total variability):   0.00%
## H^2 (total variability / sampling variability):  1.00
##
## Test for Heterogeneity:
## Q(df = 9) = 0.0000, p-val = 1.0000
##
## Model Results:
##
## estimate      se    zval    pval   ci.lb   ci.ub
##   0.1511  0.0321  4.7072  <.0001  0.0882  0.2141  ***
##
## ---
## Signif. codes:  0 '***' 0.001 '**' 0.01 '*' 0.05 '.' 0.1 ' ' 1
##
## [1] "**Output for 0.3 effect size on day 1, and 0.5 test-retest correlation (heterog., meta)**"
## [1] "*Meta-analysis: day 1 d*"
##
## Random-Effects Model (k = 10; tau^2 estimator: REML)
##
## tau^2 (estimated amount of total heterogeneity): 0.6358 (SE = 0.3151)
## tau (square root of estimated tau^2 value):      0.7973
## I^2 (total heterogeneity / total variability):   95.87%
## H^2 (total variability / sampling variability):  24.23
##
## Test for Heterogeneity:
## Q(df = 9) = 311.1326, p-val < .0001
```

```
##
## Model Results:
##
## estimate      se    zval    pval   ci.lb   ci.ub
##   1.9834  0.2585  7.6716  <.0001  1.4767  2.4901  ***
##
## ---
## Signif. codes:  0 '***' 0.001 '**' 0.01 '*' 0.05 '.' 0.1 ' ' 1
##
## [1] "*Meta-analysis: test-retest r*"
##
## Random-Effects Model (k = 10; tau^2 estimator: REML)
##
## tau^2 (estimated amount of total heterogeneity): 0 (SE = 0.0049)
## tau (square root of estimated tau^2 value):      0
## I^2 (total heterogeneity / total variability):   0.00%
## H^2 (total variability / sampling variability):  1.00
##
## Test for Heterogeneity:
## Q(df = 9) = 0.0000, p-val = 1.0000
##
## Model Results:
##
## estimate      se     zval    pval   ci.lb   ci.ub
##   0.5493  0.0321  17.1080  <.0001  0.4864  0.6122  ***
##
## ---
## Signif. codes:  0 '***' 0.001 '**' 0.01 '*' 0.05 '.' 0.1 ' ' 1
##
## [1] "**Output for 0.3 effect size on day 1, and 0.85 test-retest correlation (heterog., meta)**"
## [1] "*Meta-analysis: day 1 d*"
##
## Random-Effects Model (k = 10; tau^2 estimator: REML)
##
## tau^2 (estimated amount of total heterogeneity): 0.8546 (SE = 0.4187)
## tau (square root of estimated tau^2 value):      0.9244
## I^2 (total heterogeneity / total variability):   96.94%
## H^2 (total variability / sampling variability):  32.72
##
## Test for Heterogeneity:
## Q(df = 9) = 379.0271, p-val < .0001
##
## Model Results:
##
## estimate      se    zval    pval   ci.lb   ci.ub
##   1.9893  0.2980  6.6744  <.0001  1.4051  2.5734  ***
##
## ---
## Signif. codes:  0 '***' 0.001 '**' 0.01 '*' 0.05 '.' 0.1 ' ' 1
##
## [1] "*Meta-analysis: test-retest r*"
##
## Random-Effects Model (k = 10; tau^2 estimator: REML)
##
```

```
## tau^2 (estimated amount of total heterogeneity): 0 (SE = 0.0049)
## tau (square root of estimated tau^2 value):      0
## I^2 (total heterogeneity / total variability):   0.00%
## H^2 (total variability / sampling variability):  1.00
##
## Test for Heterogeneity:
## Q(df = 9) = 0.0000, p-val = 1.0000
##
## Model Results:
##
## estimate      se     zval    pval   ci.lb   ci.ub
##   1.2562  0.0321  39.1227  <.0001  1.1932  1.3191  ***
##
## ---
## Signif. codes:  0 '***' 0.001 '**' 0.01 '*' 0.05 '.' 0.1 ' ' 1
##
## [1] "**Output for 0.6 effect size on day 1, and 0.15 test-retest correlation (heterog., meta)**"
## [1] "*Meta-analysis: day 1 d*"
##
## Random-Effects Model (k = 10; tau^2 estimator: REML)
##
## tau^2 (estimated amount of total heterogeneity): 0.7012 (SE = 0.3745)
## tau (square root of estimated tau^2 value):      0.8374
## I^2 (total heterogeneity / total variability):   89.33%
## H^2 (total variability / sampling variability):  9.37
##
## Test for Heterogeneity:
## Q(df = 9) = 90.2017, p-val < .0001
##
## Model Results:
##
## estimate      se     zval    pval   ci.lb   ci.ub
##   4.0015  0.2819  14.1932  <.0001  3.4489  4.5541  ***
##
## ---
## Signif. codes:  0 '***' 0.001 '**' 0.01 '*' 0.05 '.' 0.1 ' ' 1
##
## [1] "*Meta-analysis: test-retest r*"
##
## Random-Effects Model (k = 10; tau^2 estimator: REML)
##
## tau^2 (estimated amount of total heterogeneity): 0 (SE = 0.0049)
## tau (square root of estimated tau^2 value):      0
## I^2 (total heterogeneity / total variability):   0.00%
## H^2 (total variability / sampling variability):  1.00
##
## Test for Heterogeneity:
## Q(df = 9) = 0.0000, p-val = 1.0000
##
## Model Results:
##
## estimate      se    zval    pval   ci.lb   ci.ub
##   0.1511  0.0321  4.7072  <.0001  0.0882  0.2141  ***
##
```

```
## ---
## Signif. codes:  0 '***' 0.001 '**' 0.01 '*' 0.05 '.' 0.1 ' ' 1
##
## [1] "**Output for 0.6 effect size on day 1, and 0.5 test-retest correlation (heterog., meta)**"
## [1] "*Meta-analysis: day 1 d*"
##
## Random-Effects Model (k = 10; tau^2 estimator: REML)
##
## tau^2 (estimated amount of total heterogeneity): 0.6988 (SE = 0.3740)
## tau (square root of estimated tau^2 value):      0.8360
## I^2 (total heterogeneity / total variability):   89.21%
## H^2 (total variability / sampling variability):  9.27
##
## Test for Heterogeneity:
## Q(df = 9) = 114.1250, p-val < .0001
##
## Model Results:
##
## estimate      se     zval    pval   ci.lb   ci.ub
##   4.0352  0.2817  14.3230  <.0001  3.4830  4.5874  ***
##
## ---
## Signif. codes:  0 '***' 0.001 '**' 0.01 '*' 0.05 '.' 0.1 ' ' 1
##
## [1] "*Meta-analysis: test-retest r*"
##
## Random-Effects Model (k = 10; tau^2 estimator: REML)
##
## tau^2 (estimated amount of total heterogeneity): 0 (SE = 0.0049)
## tau (square root of estimated tau^2 value):      0
## I^2 (total heterogeneity / total variability):   0.00%
## H^2 (total variability / sampling variability):  1.00
##
## Test for Heterogeneity:
## Q(df = 9) = 0.0000, p-val = 1.0000
##
## Model Results:
##
## estimate      se     zval    pval   ci.lb   ci.ub
##   0.5493  0.0321  17.1080  <.0001  0.4864  0.6122  ***
##
## ---
## Signif. codes:  0 '***' 0.001 '**' 0.01 '*' 0.05 '.' 0.1 ' ' 1
##
## [1] "**Output for 0.6 effect size on day 1, and 0.85 test-retest correlation (heterog., meta)**"
## [1] "*Meta-analysis: day 1 d*"
##
## Random-Effects Model (k = 10; tau^2 estimator: REML)
##
## tau^2 (estimated amount of total heterogeneity): 0.6162 (SE = 0.3346)
## tau (square root of estimated tau^2 value):      0.7850
## I^2 (total heterogeneity / total variability):   87.92%
## H^2 (total variability / sampling variability):  8.28
##
```

```
## Test for Heterogeneity:
## Q(df = 9) = 108.0341, p-val < .0001
##
## Model Results:
##
## estimate      se     zval    pval   ci.lb   ci.ub
##   4.0217  0.2665  15.0921  <.0001  3.4994  4.5440  ***
##
## ---
## Signif. codes:  0 '***' 0.001 '**' 0.01 '*' 0.05 '.' 0.1 ' ' 1
##
## [1] "*Meta-analysis: test-retest r*"
##
## Random-Effects Model (k = 10; tau^2 estimator: REML)
##
## tau^2 (estimated amount of total heterogeneity): 0 (SE = 0.0049)
## tau (square root of estimated tau^2 value):      0
## I^2 (total heterogeneity / total variability):   0.00%
## H^2 (total variability / sampling variability):  1.00
##
## Test for Heterogeneity:
## Q(df = 9) = 0.0000, p-val = 1.0000
##
## Model Results:
##
## estimate      se     zval    pval   ci.lb   ci.ub
##   1.2562  0.0321  39.1227  <.0001  1.1932  1.3191  ***
##
## ---
## Signif. codes:  0 '***' 0.001 '**' 0.01 '*' 0.05 '.' 0.1 ' ' 1
```

```
  colnames(allds) <- c("day1size","sim_r","d","SE(d)")
  colnames(allrs) <- c("day1size","sim_r","rz","SE(rz)","r","SE(r)")

  allds_meta_het <- allds
  allrs_meta_het <- allrs
```

```
# generate pq1d at the child level dividing by group-based sd
dt$Keyname=paste("day1size",dt$day1size,"r",dt$r,"study",dt$study)
dt1=merge(effects[,c("group_PQ1","group_SD1","r_calc","N","Keyname",
                     "d_calc","d_var_calc")],dt, by="Keyname")
dt1$d1=dt1$PQ1/dt1$group_SD1

allb_d1s=allb_rs=NULL
for(thisS in myS) for(thisR in myR){

  print(paste("**Output for",thisS,"effect size on day 1, and", thisR,
             "test-retest correlation (heterog., mega)**"))
  #print(c(thisS,thisR))

    print(paste("*Mega-analysis: day 1 d*"))

  mega_d1 = lmer(d1 ~ 1 + (1 | Keyname),data=dt1,
```

```r
                subset=c(day1size==thisS & r==thisR))
  print(mega_d1)

  ran.variance.d <- as.data.frame(VarCorr(mega_d1))
  allb_d1s=rbind(allb_d1s,
              cbind(thisS, thisR,
                    summary(mega_d1)$coefficients["(Intercept)","Estimate"],
                    summary(mega_d1)$coefficients["(Intercept)","Std. Error"],
                    100*round(ran.variance.d$vcov[1]/
                                (ran.variance.d$vcov[1]+ran.variance.d$vcov[2]),2)))

  print(paste("*Mega-analysis: test-retest r*"))

  mega_r =lmer(PQ2 ~ PQ1 + (1 + PQ1 | study),data=dt1,
              subset=c(day1size==thisS & r==thisR),
              control=lmerControl(optimizer="bobyqa"))
  print(mega_r)

 ran.variance.r <- as.data.frame(VarCorr(mega_r))
  allb_rs=rbind(allb_rs,
              cbind(thisS, thisR, summary(mega_r)$coefficients["(Intercept)","Estimate"],
                    summary(mega_r)$coefficients["PQ1","Estimate"],
                    summary(mega_r)$coefficients["(Intercept)","Std. Error"],
                    summary(mega_r)$coefficients["PQ1","Std. Error"],
                    100*round(ran.variance.r$vcov[1]/
 (ran.variance.r$vcov[1]+ran.variance.r$vcov[2]+ran.variance.r$vcov[4]),2),
                    100*round(ran.variance.r$vcov[2]/
(ran.variance.r$vcov[1]+ran.variance.r$vcov[2]+ran.variance.r$vcov[4]),2)))


}
```

Mega-analyses

```
## [1] "**Output for 0 effect size on day 1, and 0.15 test-retest correlation (heterog., mega)**"
## [1] "*Mega-analysis: day 1 d*"
## Linear mixed model fit by REML ['lmerModLmerTest']
## Formula: d1 ~ 1 + (1 | Keyname)
##    Data: dt1
##  Subset: c(day1size == thisS & r == thisR)
## REML criterion at convergence: 2879.302
## Random effects:
##  Groups   Name        Std.Dev.
##  Keyname  (Intercept) 0.7904
##  Residual             1.0000
## Number of obs: 1000, groups:  Keyname, 10
## Fixed Effects:
## (Intercept)
##     0.01801
## [1] "*Mega-analysis: test-retest r*"
## Linear mixed model fit by REML ['lmerModLmerTest']
## Formula: PQ2 ~ PQ1 + (1 + PQ1 | study)
##    Data: dt1
##  Subset: c(day1size == thisS & r == thisR)
```

```
## REML criterion at convergence: -1009.855
## Random effects:
##  Groups   Name        Std.Dev. Corr
##  study    (Intercept) 0.1147
##           PQ1         0.1940   -0.06
##  Residual             0.1414
## Number of obs: 1000, groups:  study, 10
## Fixed Effects:
## (Intercept)          PQ1
##   -0.008502     0.160195
## [1] "**Output for 0 effect size on day 1, and 0.5 test-retest correlation (heterog., mega)**"
## [1] "*Mega-analysis: day 1 d*"
## Linear mixed model fit by REML ['lmerModLmerTest']
## Formula: d1 ~ 1 + (1 | Keyname)
##    Data: dt1
##  Subset: c(day1size == thisS & r == thisR)
## REML criterion at convergence: 2881.317
## Random effects:
##  Groups   Name        Std.Dev.
##  Keyname  (Intercept) 0.8854
##  Residual             1.0000
## Number of obs: 1000, groups:  Keyname, 10
## Fixed Effects:
## (Intercept)
##    -0.03765
## [1] "*Mega-analysis: test-retest r*"
## Linear mixed model fit by REML ['lmerModLmerTest']
## Formula: PQ2 ~ PQ1 + (1 + PQ1 | study)
##    Data: dt1
##  Subset: c(day1size == thisS & r == thisR)
## REML criterion at convergence: -1214.989
## Random effects:
##  Groups   Name        Std.Dev. Corr
##  study    (Intercept) 0.0632
##           PQ1         0.1860   -0.06
##  Residual             0.1281
## Number of obs: 1000, groups:  study, 10
## Fixed Effects:
## (Intercept)          PQ1
##    0.006315     0.512938
## [1] "**Output for 0 effect size on day 1, and 0.85 test-retest correlation (heterog., mega)**"
## [1] "*Mega-analysis: day 1 d*"
## Linear mixed model fit by REML ['lmerModLmerTest']
## Formula: d1 ~ 1 + (1 | Keyname)
##    Data: dt1
##  Subset: c(day1size == thisS & r == thisR)
## REML criterion at convergence: 2880.948
## Random effects:
##  Groups   Name        Std.Dev.
##  Keyname  (Intercept) 0.8672
##  Residual             1.0000
## Number of obs: 1000, groups:  Keyname, 10
## Fixed Effects:
## (Intercept)
```

```
##     0.02762
## [1] "*Mega-analysis: test-retest r*"
## Linear mixed model fit by REML ['lmerModLmerTest']
## Formula: PQ2 ~ PQ1 + (1 + PQ1 | study)
##    Data: dt1
##  Subset: c(day1size == thisS & r == thisR)
## REML criterion at convergence: -2217.862
## Random effects:
##  Groups   Name        Std.Dev. Corr
##  study    (Intercept) 0.02537
##           PQ1         0.10742  -0.18
##  Residual             0.07780
## Number of obs: 1000, groups:  study, 10
## Fixed Effects:
## (Intercept)          PQ1
##     0.00725      0.83623
## [1] "**Output for 0.3 effect size on day 1, and 0.15 test-retest correlation (heterog., mega)**"
## [1] "*Mega-analysis: day 1 d*"
## Linear mixed model fit by REML ['lmerModLmerTest']
## Formula: d1 ~ 1 + (1 | Keyname)
##    Data: dt1
##  Subset: c(day1size == thisS & r == thisR)
## REML criterion at convergence: 2880.247
## Random effects:
##  Groups   Name        Std.Dev.
##  Keyname  (Intercept) 0.8336
##  Residual             1.0000
## Number of obs: 1000, groups:  Keyname, 10
## Fixed Effects:
## (Intercept)
##       2.045
## [1] "*Mega-analysis: test-retest r*"
## Linear mixed model fit by REML ['lmerModLmerTest']
## Formula: PQ2 ~ PQ1 + (1 + PQ1 | study)
##    Data: dt1
##  Subset: c(day1size == thisS & r == thisR)
## REML criterion at convergence: -989.5069
## Random effects:
##  Groups   Name        Std.Dev. Corr
##  study    (Intercept) 0.1344
##           PQ1         0.2170   -0.60
##  Residual             0.1429
## Number of obs: 1000, groups:  study, 10
## Fixed Effects:
## (Intercept)          PQ1
##      0.2560       0.1696
## [1] "**Output for 0.3 effect size on day 1, and 0.5 test-retest correlation (heterog., mega)**"
## [1] "*Mega-analysis: day 1 d*"
## Linear mixed model fit by REML ['lmerModLmerTest']
## Formula: d1 ~ 1 + (1 | Keyname)
##    Data: dt1
##  Subset: c(day1size == thisS & r == thisR)
## REML criterion at convergence: 2879.569
## Random effects:
```

```
##  Groups   Name        Std.Dev.
##  Keyname  (Intercept) 0.8024  
##  Residual             1.0000  
## Number of obs: 1000, groups:  Keyname, 10
## Fixed Effects:
## (Intercept)  
##       1.998  
## [1] "*Mega-analysis: test-retest r*"
## Linear mixed model fit by REML ['lmerModLmerTest']
## Formula: PQ2 ~ PQ1 + (1 + PQ1 | study)
##    Data: dt1
##  Subset: c(day1size == thisS & r == thisR)
## REML criterion at convergence: -1187.572
## Random effects:
##  Groups   Name        Std.Dev. Corr 
##  study    (Intercept) 0.08935       
##           PQ1         0.18368  -0.52
##  Residual             0.12964       
## Number of obs: 1000, groups:  study, 10
## Fixed Effects:
## (Intercept)          PQ1  
##      0.1474       0.4953  
## [1] "**Output for 0.3 effect size on day 1, and 0.85 test-retest correlation (heterog., mega)**"
## [1] "*Mega-analysis: day 1 d*"
## Linear mixed model fit by REML ['lmerModLmerTest']
## Formula: d1 ~ 1 + (1 | Keyname)
##    Data: dt1
##  Subset: c(day1size == thisS & r == thisR)
## REML criterion at convergence: 2882.222
## Random effects:
##  Groups   Name        Std.Dev.
##  Keyname  (Intercept) 0.9316  
##  Residual             1.0000  
## Number of obs: 1000, groups:  Keyname, 10
## Fixed Effects:
## (Intercept)  
##       2.004  
## [1] "*Mega-analysis: test-retest r*"
## Linear mixed model fit by REML ['lmerModLmerTest']
## Formula: PQ2 ~ PQ1 + (1 + PQ1 | study)
##    Data: dt1
##  Subset: c(day1size == thisS & r == thisR)
## REML criterion at convergence: -2231.168
## Random effects:
##  Groups   Name        Std.Dev. Corr 
##  study    (Intercept) 0.02953       
##           PQ1         0.10498  -0.85
##  Residual             0.07753       
## Number of obs: 1000, groups:  study, 10
## Fixed Effects:
## (Intercept)          PQ1  
##     0.05401      0.82614  
## [1] "**Output for 0.6 effect size on day 1, and 0.15 test-retest correlation (heterog., mega)**"
## [1] "*Mega-analysis: day 1 d*"
```

```
## Linear mixed model fit by REML ['lmerModLmerTest']
## Formula: d1 ~ 1 + (1 | Keyname)
##    Data: dt1
##  Subset: c(day1size == thisS & r == thisR)
## REML criterion at convergence: 2881.3
## Random effects:
##  Groups   Name        Std.Dev.
##  Keyname  (Intercept) 0.8845
##  Residual             1.0000
## Number of obs: 1000, groups:  Keyname, 10
## Fixed Effects:
## (Intercept)
##       4.037
## [1] "*Mega-analysis: test-retest r*"
## Linear mixed model fit by REML ['lmerModLmerTest']
## Formula: PQ2 ~ PQ1 + (1 + PQ1 | study)
##    Data: dt1
##  Subset: c(day1size == thisS & r == thisR)
## REML criterion at convergence: -964.8288
## Random effects:
##  Groups   Name        Std.Dev. Corr
##  study    (Intercept) 0.1794
##           PQ1         0.2097   -0.79
##  Residual             0.1446
## Number of obs: 1000, groups:  study, 10
## Fixed Effects:
## (Intercept)          PQ1
##      0.5106       0.1607
## [1] "**Output for 0.6 effect size on day 1, and 0.5 test-retest correlation (heterog., mega)**"
## [1] "*Mega-analysis: day 1 d*"
## Linear mixed model fit by REML ['lmerModLmerTest']
## Formula: d1 ~ 1 + (1 | Keyname)
##    Data: dt1
##  Subset: c(day1size == thisS & r == thisR)
## REML criterion at convergence: 2880.807
## Random effects:
##  Groups   Name        Std.Dev.
##  Keyname  (Intercept) 0.8604
##  Residual             1.0000
## Number of obs: 1000, groups:  Keyname, 10
## Fixed Effects:
## (Intercept)
##       4.067
## [1] "*Mega-analysis: test-retest r*"
## Linear mixed model fit by REML ['lmerModLmerTest']
## Formula: PQ2 ~ PQ1 + (1 + PQ1 | study)
##    Data: dt1
##  Subset: c(day1size == thisS & r == thisR)
## REML criterion at convergence: -1180.182
## Random effects:
##  Groups   Name        Std.Dev. Corr
##  study    (Intercept) 0.1367
##           PQ1         0.1887   -0.88
##  Residual             0.1303
```

```
## Number of obs: 1000, groups:  study, 10
## Fixed Effects:
## (Intercept)          PQ1
##      0.2963       0.5171
## [1] "**Output for 0.6 effect size on day 1, and 0.85 test-retest correlation (heterog., mega)**"
## [1] "*Mega-analysis: day 1 d*"
## Linear mixed model fit by REML ['lmerModLmerTest']
## Formula: d1 ~ 1 + (1 | Keyname)
##    Data: dt1
##  Subset: c(day1size == thisS & r == thisR)
## REML criterion at convergence: 2879.535
## Random effects:
##  Groups   Name        Std.Dev.
##  Keyname  (Intercept) 0.8008
##  Residual             1.0000
## Number of obs: 1000, groups:  Keyname, 10
## Fixed Effects:
## (Intercept)
##       4.052
## [1] "*Mega-analysis: test-retest r*"
## Linear mixed model fit by REML ['lmerModLmerTest']
## Formula: PQ2 ~ PQ1 + (1 + PQ1 | study)
##    Data: dt1
##  Subset: c(day1size == thisS & r == thisR)
## REML criterion at convergence: -2106.779
## Random effects:
##  Groups   Name        Std.Dev. Corr
##  study    (Intercept) 0.03122
##           PQ1         0.05335  -0.75
##  Residual             0.08276
## Number of obs: 1000, groups:  study, 10
## Fixed Effects:
## (Intercept)          PQ1
##     0.08225      0.86373
```

```
colnames(allb_d1s)<-c("day1size", "r","b0","SE(b0)","REvarexplained")

colnames(allb_rs)<-c("day1size", "r","b0","b1","SE(b0)","SE(b1)",
                     "REvarexplained_int","REvarexplained_slope")

  allds_mega_het <- allb_d1s
  allrs_mega_het <- allb_rs
```

Appendix B: Hybrid re-analyses with intermediate number of data points

In this Appendix, we show that there is little effect to replacing some or all of the studies with generated data. To do this, we start with results where all studies are represented by their natural, original data (from the Cristia et al study). We then replace one, two, … thirteen studies (picked at random) with synthetic data. For simplicity, we only show this once (we have fixed our random seed), but we checked that this was stable regardless of which studies were picked. Readers can check for themselves by changing the seed.

```r
#set.seed(42) #uncomment & change number to see a different draw

# we fix naming of variables between pairs2 and pairs.sample
colnames(pairs.sample)<-c("pq1","pq2","study","sd1","sd2","d1","d2")

# we remove extra columns from pairs2, to be able to rbind the two in the loop below
pairs2=pairs2[,colnames(pairs.sample)]

#start the tables
allb_d1s=allb_rs=NULL

#original case: all studies represented by their natural data
nGen=0

## add the d values
mega_d1 = lmer(d1 ~ 1 + (1 | study),data=pairs2)
  ran.variance.d <- as.data.frame(VarCorr(mega_d1))
  allb_d1s=rbind(allb_d1s,
              cbind(nGen, summary(mega_d1)$coefficients["(Intercept)","Estimate"],
                    summary(mega_d1)$coefficients["(Intercept)","Std. Error"],
                    100*round(ran.variance.d$vcov[1]/
 (ran.variance.d$vcov[1]+ran.variance.d$vcov[2]),2)))

## add the r values
mega_r =lmer(pq2 ~ pq1 + (1 + pq1 | study),data=pairs2,
             control=lmerControl(optimizer="bobyqa", optCtrl=list(maxfun=2e5)))
 ran.variance.r <- as.data.frame(VarCorr(mega_r))
  allb_rs=rbind(allb_rs,
              cbind(nGen, summary(mega_r)$coefficients["(Intercept)","Estimate"],
                    summary(mega_r)$coefficients["pq1","Estimate"],
                    summary(mega_r)$coefficients["(Intercept)","Std. Error"],
                    summary(mega_r)$coefficients["pq1","Std. Error"],
                    100*round(ran.variance.r$vcov[1]/
 (ran.variance.r$vcov[1]+ran.variance.r$vcov[2]+ran.variance.r$vcov[4]),2),
                    100*round(ran.variance.r$vcov[2]/
(ran.variance.r$vcov[1]+ran.variance.r$vcov[2]+ran.variance.r$vcov[4]),2)))


for(nGen in 1:length(levels(factor(pairs2$study)))) {

    #pick studies to be replaced with synthetic data
    to_replace=sample(levels(factor(pairs2$study)),nGen)

    new_pairs<-rbind(
      pairs2[!(pairs2$study %in% to_replace),],
      #from pairs2, take all studies EXCEPT those to be replaced
      pairs.sample[(pairs.sample$study %in% to_replace),]
      #from pairs.sample, take ONLY those to be replaced
    )

    mega_d1 = lmer(d1 ~ 1 + (1 | study),data=new_pairs)
    ran.variance.d <- as.data.frame(VarCorr(mega_d1))
    allb_d1s=rbind(allb_d1s,
```

```
                    cbind(nGen,
                          summary(mega_d1)$coefficients["(Intercept)","Estimate"],
                          summary(mega_d1)$coefficients["(Intercept)","Std. Error"],
                          100*round(ran.variance.d$vcov[1]/
(ran.variance.d$vcov[1]+ran.variance.d$vcov[2]),2)))


    mega_r =lmer(pq2 ~ pq1 + (1 + pq1 | study),data=new_pairs,
                  control=lmerControl(optimizer="bobyqa", optCtrl=list(maxfun=2e5)))
    ran.variance.r <- as.data.frame(VarCorr(mega_r))

    allb_rs=rbind(allb_rs,
                   cbind(nGen, summary(mega_r)$coefficients["(Intercept)","Estimate"],
                         summary(mega_r)$coefficients["pq1","Estimate"],
                         summary(mega_r)$coefficients["(Intercept)","Std. Error"],
                         summary(mega_r)$coefficients["pq1","Std. Error"],
                         100*round(ran.variance.r$vcov[1]/
(ran.variance.r$vcov[1]+ran.variance.r$vcov[2]+ran.variance.r$vcov[4]),2),
                         100*round(ran.variance.r$vcov[2]/
(ran.variance.r$vcov[1]+ran.variance.r$vcov[2]+ran.variance.r$vcov[4]),2)))

}

colnames(allb_d1s)<-c("Ngenerated","b0","SE(b0)","REvarexplained")

colnames(allb_rs)<-c("Ngenerated","b0","b1","SE(b0)","SE(b1)",
                     "REvarexplained_int","REvarexplained_slope")
```

Results of hybrid analyses for day 1 d

We see some variation in the Cohen's d estimated from these hybrid datasets, in the order of maximally .04. Notice additionally that there is no clear direction whereby more synthetic data leads to higher or lower estimates. The standard errors are also extremely stable, with maximally .01 variation. As for the percentage of variance attributed to random slopes, this varies more markedly, with the maximum variation being 6% between the case with the maximum and that with the minimum values. Nonetheless, here again, no systematic direction of change is observed.

```
allb_d1s[,2:3]<-round(allb_d1s[,2:3],3)
kable(allb_d1s,caption="Results of mixed models fit to hybrid dataset, with some real data replaced by g
```

Table 1: Results of mixed models fit to hybrid dataset, with some real data replaced by generated data, predicting the first day's PQ. Ngenerated indicates the number of studies that have been replaced with generated data (0 indicates no studies replaced, i.e., mega-analytic results in the main paper). b0 shows the beta for the intercept (here Cohen's d for PQ1). SE(b0) shows the standard error of that beta. REvarexplained shows the percentage of variance explained by random intercepts per study.

Ngenerated	b0	SE(b0)	REvarexplained
0	0.421	0.238	41
1	0.429	0.239	41
2	0.392	0.240	41
3	0.436	0.249	43

Ngenerated	b0	SE(b0)	REvarexplained
4	0.437	0.249	42
5	0.416	0.240	40
6	0.405	0.235	39
7	0.411	0.232	38
8	0.397	0.231	37
9	0.398	0.241	41
10	0.407	0.242	42
11	0.391	0.241	40
12	0.418	0.242	41
13	0.409	0.244	40

Results of hybrid analyses for test-retest r

Here we repeat the analyses of the hybrid datasets, focusing on the test-retest correlations. Conclusions are exactly as for the analyses of day 1 PQ: effects of how much data is generated are small and unstable; the largest effects can be seen in percent variance attributed to random effects structure.

```
allb_rs[,2:5]<-round(allb_rs[,2:5],3)
allb_rs=allb_rs[,c(1:2,4,3,5:7)] #swapping order of b1 and SE(b0)
kable(allb_rs,caption="Results of mixed models fit to hybrid dataset, with some real data replaced by g
```

Table 2: Results of mixed models fit to hybrid dataset, with some real data replaced by generated data, predicting the association between day 1 and day 2 PQ. Ngenerated indicates the number of studies that have been replaced with generated data (0 indicates no studies replaced, i.e., mega-analytic results in the main paper). b0 shows the beta for the intercept (here PQ2). SE(b0) shows the standard error of that beta. b1 shows the beta for the PQ1 predictor. SE(b1) shows the standard error of that beta. REvarexplained_int shows the percentage of variance explained by random intercepts per study. REvarexplained_slope shows the percentage of variance explained by random slopes for PQ1 per study.

Ngenerated	b0	SE(b0)	b1	SE(b1)	REvarexplained_int	REvarexplained_slope
0	0.066	0.041	0.095	0.077	19	42
1	0.074	0.043	0.083	0.075	22	39
2	0.069	0.041	0.091	0.076	19	41
3	0.070	0.041	0.104	0.077	19	41
4	0.072	0.042	0.099	0.076	20	40
5	0.066	0.044	0.106	0.078	21	40
6	0.071	0.043	0.093	0.076	21	39
7	0.071	0.044	0.074	0.069	24	34
8	0.066	0.045	0.094	0.074	23	36
9	0.073	0.042	0.093	0.074	20	38
10	0.063	0.042	0.107	0.075	20	38
11	0.060	0.042	0.095	0.071	22	35
12	0.073	0.044	0.086	0.070	24	34
13	0.072	0.045	0.090	0.071	24	33

Appendix C: Varying number of participants per experiment N

```
library("simstudy")


#random noise for each study
simulation_unsatisfactory <- TRUE
while(simulation_unsatisfactory==TRUE){

try.random.pq1 <- rnorm(10,0,stdevpq1study)
#try.random.r <- rnorm(10,0,stdevrstudy)
try.random.z <- rnorm(10,0,stdevzstudy)

sim.d <- mean(try.random.pq1)/stdevpq1study
sim.d.sd <- sd(try.random.pq1)/stdevpq1study
#sim.r <- mean(try.random.r)
#sim.r.sd <- sd(try.random.r)
sim.z <- mean(try.random.z)
sim.z.sd <- sd(try.random.z)/stdevzstudy

simulation_unsatisfactory = !(abs(sim.d)<0.01 &
                              #abs(sim.z)<0.01*1000 &
                              abs(sim.z)<0.01 &
                              abs(sim.d.sd)>1 &
                              #abs(sim.z.sd)> 0.05/1000
                              abs(sim.z.sd)> 1
                                )
              }

my.random.pq1 <- try.random.pq1
#my.random.r <- try.random.r
my.random.z <- try.random.z
#my.random.r <- tanh(my.random.z)

#We vary the amount of participants per study N:
N <- round(runif(10,10,200))
numpart <- as.data.frame(cbind(1:10,N))
colnames(numpart) <- c("Study", "#Part.")
kable(numpart,caption="Number of participants per study")
```

Table 3: Number of participants per study

Study	#Part.
1	115
2	154
3	189
4	17
5	176
6	11
7	44
8	14
9	14
10	26

```
# Case 1 (Homogeneus data): studies are essentially replications
dt=NULL
##





##
for(thisS in myS){

  for(thisz in myZ){
    thisr <- tanh(thisz)
      mu <- c(thisS, thisS)  #thisS*thisr
    #Cz <- matrix(c(1, thisz, thisz, 1), nrow = 2)
    for(i in 1:10){ #10 studies
      #generate the data for this study
      #control loop
       simulation_unsatisfactory <- TRUE
      while(simulation_unsatisfactory==TRUE){
        try.this  <- as.data.frame(
genCorData(N[i], mu = mu+my.random.pq1[i], sigma = c(stdevpq1, stdevpq1), corMatrix =  matrix(c(1,tanh(
      )
        sim.cor <- cor.test(try.this$pref1,try.this$pref2)$estimate
        sim.mean <- mean(try.this$pref1)
        sim.sd <- sd(try.this$pref1)
        simulation_unsatisfactory = !(abs(sim.cor-tanh(thisz + my.random.z[i]))/tanh(thisz)<0.005 &
                                        abs(sim.mean - (thisS + my.random.pq1[i]))<0.1  &
                                        abs(sim.sd-stdevpq1)<0.05)

              }
       temp <- try.this
      #thisstudy= cbind(try.this,as.character(exp_full$study[i]),sd1[i],sd1[2])
      #
      #  temp <- genCorData(100, mu = mu+my.random.pq1[i], sigma = c(stdevpq1, stdevpq1), corMatrix = C
      # #temp <- genCorData(100, mu = rep(thisS+my.random.pq1[i],2), sigma = stdevpq1, rho = thisr + my

      dt=rbind(dt,
               cbind(thisS,thisr,i,temp))
    }

  }
}

colnames(dt)<-c("day1size", "r","study","childID","PQ1","PQ2")

write.csv(dt,"homogeneous-nvar.csv",row.names = F)
```

```
read.csv("homogeneous-nvar.csv")->dt




#we need, at the level of the study,:
#pq1, sd1, r
aggregate(dt$PQ1,by=list(dt$day1size,dt$r,dt$study),mean)->means
```

```
aggregate(dt$PQ1,by=list(dt$day1size,dt$r,dt$study),sd)->sds
cbind(by(dt[,c("PQ1","PQ2")],list(dt$day1size,dt$r,dt$study),function(x) cor(x$PQ1,x$PQ2,method="p")))-

effects<-cbind(means,sds$x,cors)
colnames(effects)<-c("day1size","r","study","group_PQ1","group_SD1","r_calc")

effects$N<-numpart[effects$study,]$`#Part.`
effects$Keyname<-paste("day1size",effects$day1size,"r",effects$r,"study",effects$study)
```

```
library(metafor)

effects$d_calc=effects$group_PQ1/effects$group_SD1
effects$d_var_calc <- (1 / effects$N) + (effects$d_calc ^ 2 / (2 * effects$N))

random.meta.homo.d1 <- NULL
random.meta.homo.r<-  NULL
count <- 0

allds=allrs=NULL
for(thisS in myS) for(thisR in myR){
  rma(yi=effects$d_calc, vi=effects$d_var_calc, ni=effects$N,slab=effects$study,method="REML",subset=c(
      control=list(maxiter=1000))->meta_d1
  allds=rbind(allds,cbind(thisS,thisR,meta_d1$b,meta_d1$se))

this.ranef <-as.data.frame(ranef(meta_d1))
this.ranef$myS <- thisS
this.ranef$myR <- thisR
random.meta.homo.d1 <- if(count==0){this.ranef}else{rbind(random.meta.homo.d1,this.ranef)}

  rma(ri=effects$r_calc, ni=effects$N,measure="ZCOR",slab=effects$study,method="REML",subset=c(effects$
      control=list(maxiter=1000))->meta_r
  allrs=rbind(allrs,cbind(thisS,thisR,meta_r$b,meta_r$se, tanh(meta_r$b), (1-tanh(meta_r$b)^2)*meta_r$s

this.ranef <-as.data.frame(ranef(meta_r))
this.ranef$myS <- thisS
this.ranef$myR <- thisR
#transform rz to r
this.ranef$r <- (1-thisR^2)*this.ranef$pred
this.ranef$SEr <- (1-thisR^2)*this.ranef$se


random.meta.homo.r <- if(count==0){this.ranef}else{rbind(random.meta.homo.r,this.ranef)}


    count<-count+1

  }
  colnames(allds) <- c("day1size","sim_r","d","SE(d)")
  colnames(allrs) <- c("day1size","sim_r","rz","SE(rz)","r","SE(r)")

  allds_meta_hom <- allds
  allrs_meta_hom <- allrs
```

```
library(lme4)
library(lmerTest)

# generate pq1d at the child level dividing by group-based sd
dt$Keyname=paste("day1size",dt$day1size,"r",dt$r,"study",dt$study)
dt1=merge(effects[,c("group_PQ1","group_SD1","r_calc","N","Keyname","d_calc","d_var_calc")],dt, by="Keyn
dt1$d1=dt1$PQ1/dt1$group_SD1

allb_d1s=allb_rs=NULL


random.mega.homo.d1 <- NULL
random.mega.homo.r<-  NULL
count <- 0

for(thisS in myS) for(thisR in myR){

 # print(c(thisS,thisR))

  mega_d1 = lmer(d1 ~ 1 + (1 | study),data=dt1,subset=c(day1size==thisS & r==thisR),control=lmerControl
  ran.variance.d <- as.data.frame(VarCorr(mega_d1))
  allb_d1s=rbind(allb_d1s,
              cbind(thisS, thisR, summary(mega_d1)$coefficients["(Intercept)","Estimate"],summary(mega_c


this.ranef <-as.data.frame(ranef(mega_d1))
this.ranef$myS <- thisS
this.ranef$myR <- thisR

random.mega.homo.d1 <- if(count==0){this.ranef}else{rbind(random.mega.homo.d1,this.ranef)}


  mega_r =lmer(PQ2 ~ PQ1 + (1 + PQ1 | study),data=dt1,subset=c(day1size==thisS & r==thisR),control=lmer(



 ran.variance.r <- as.data.frame(VarCorr(mega_r))
  allb_rs=rbind(allb_rs,
              cbind(thisS, thisR, summary(mega_r)$coefficients["(Intercept)","Estimate"], summary(mega_r


this.ranef <-as.data.frame(ranef(mega_r))
this.ranef$myS <- thisS
this.ranef$myR <- thisR

random.mega.homo.r <- if(count==0){this.ranef}else{rbind(random.mega.homo.r,this.ranef)}

 count <- count + 1
}

colnames(allb_d1s)<-c("day1size","r","b0","SE(b0)","REvarexplained")

colnames(allb_rs)<-c("day1size","r","b0","b1","SE(b0)","SE(b1)","REvarexplained_int","REvarexplained_slo
```

```
allds_mega_hom <- allb_d1s
allrs_mega_hom <- allb_rs
```

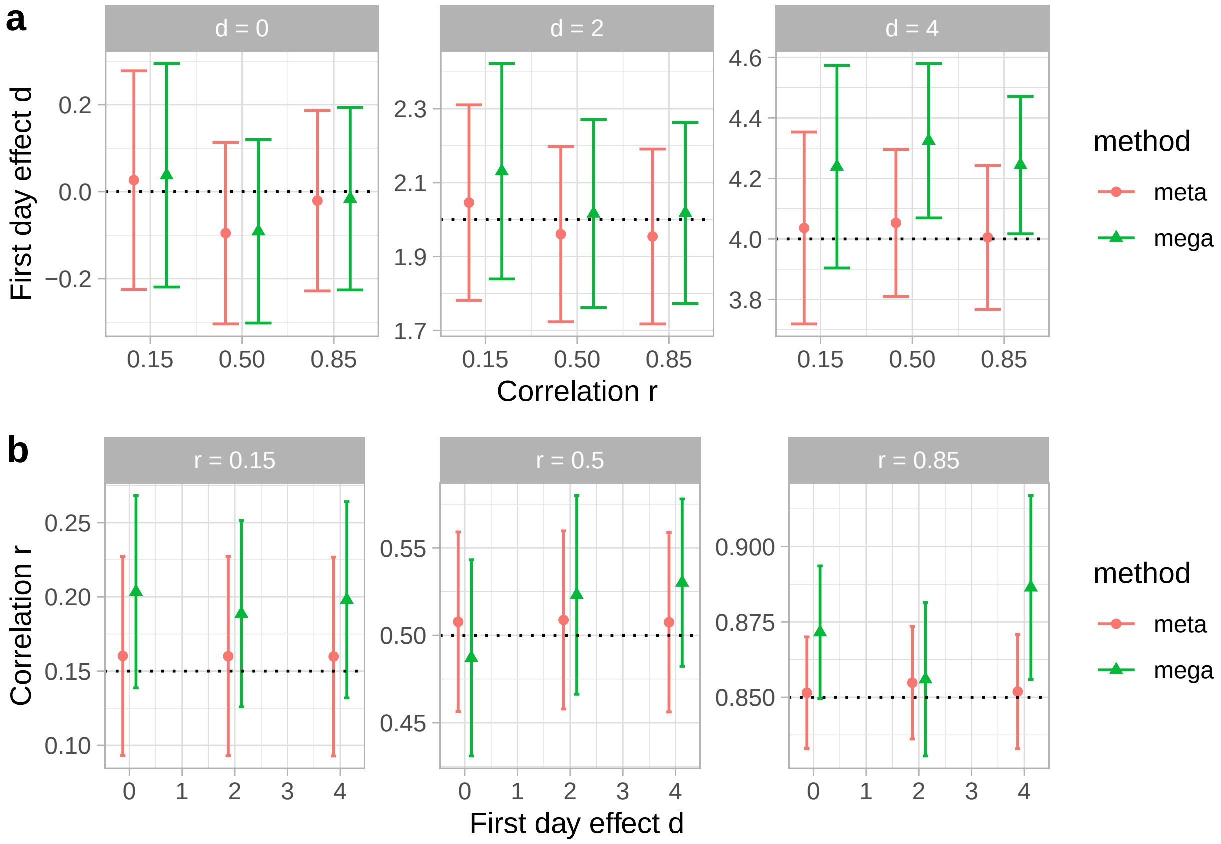

Figure 1: Fixed effects for synthetic data with varying participants' number in the analysis of effect size in the first day of testing (a) and of the correlation size across testing days (b), according to two estimation methods: a meta-analysis (meta); a regression with the true individual-level data (mega). In (a), we plot calculated d as a function of true effect size d in the data generation, and each horizontal panel represents different values of true correlation r. In (b), we plot calculated r as a function of parameter r in data generation, and each horizontal panel represents different values of effect size d. Error bars represent standard errors for the fitted values. Horizontal dotted lines represent the true value of the parameter.

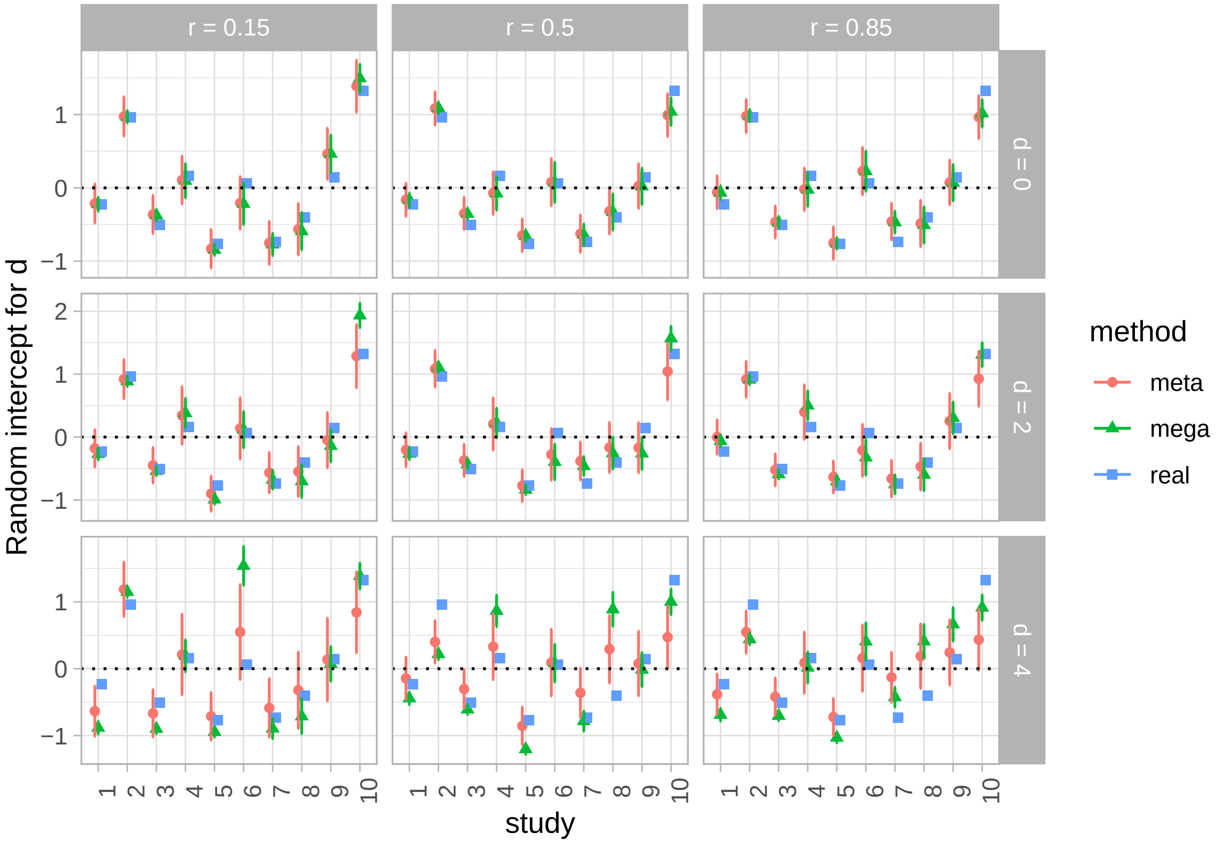

Figure 2: Random intercepts per study for synthetic data with varying participants' number in the analysis of effects in the first day of testing, according to two estimation methods: a meta-analysis (meta); a regression with the true individual-level data (mega). In each panel, we plot calculated d as a function of the true values of d and r in the data generation. Error bars represent standard errors for the fitted values.

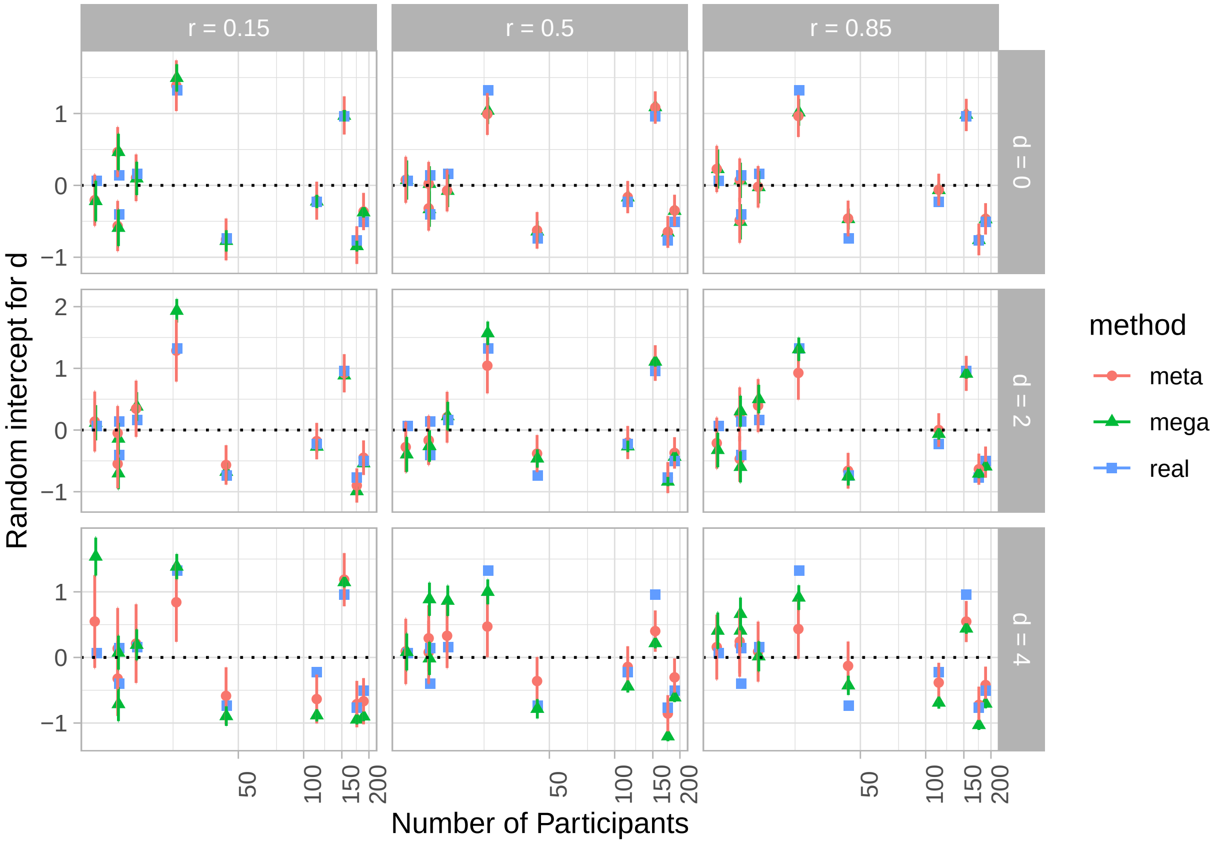

Figure 3: Random intercepts per study for synthetic data as a function of participants' number in the analysis of effects in the first day of testing, according to two estimation methods: a meta-analysis (meta); a regression with the true individual-level data (mega). In each panel, we plot calculated d as a function of the true values of d and r in the data generation. Error bars represent standard errors for the fitted values. We can see how both mega- and meta-analysis improve their predictions as the number of participants increases.

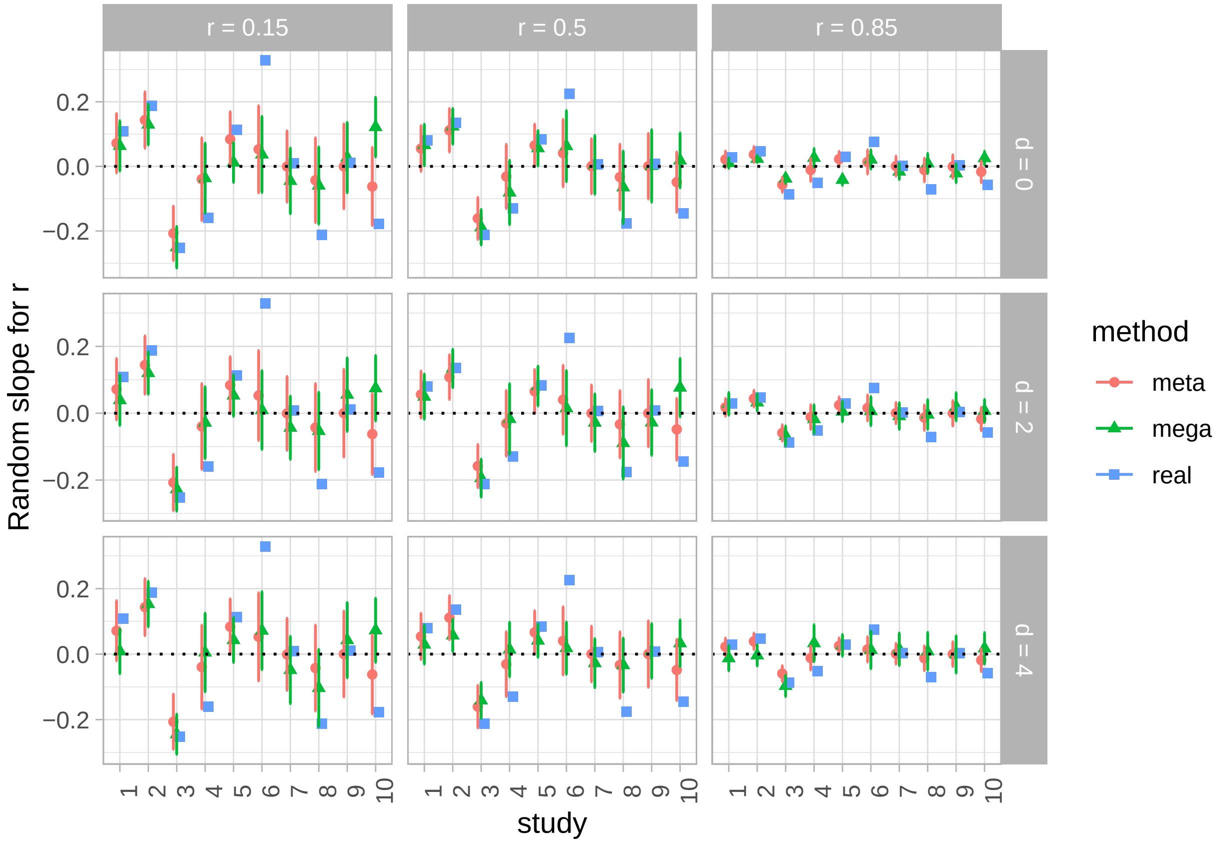

Figure 4: Random slopes per study for synthetic data with varying participants' number in the analysis of the correlation between days of testing, according to two estimation methods: a meta-analysis (meta); a regression with the true individual-level data (mega). In each panel, we plot calculated d as a function of the true values of d and r in the data generation. Error bars represent standard errors for the fitted values.

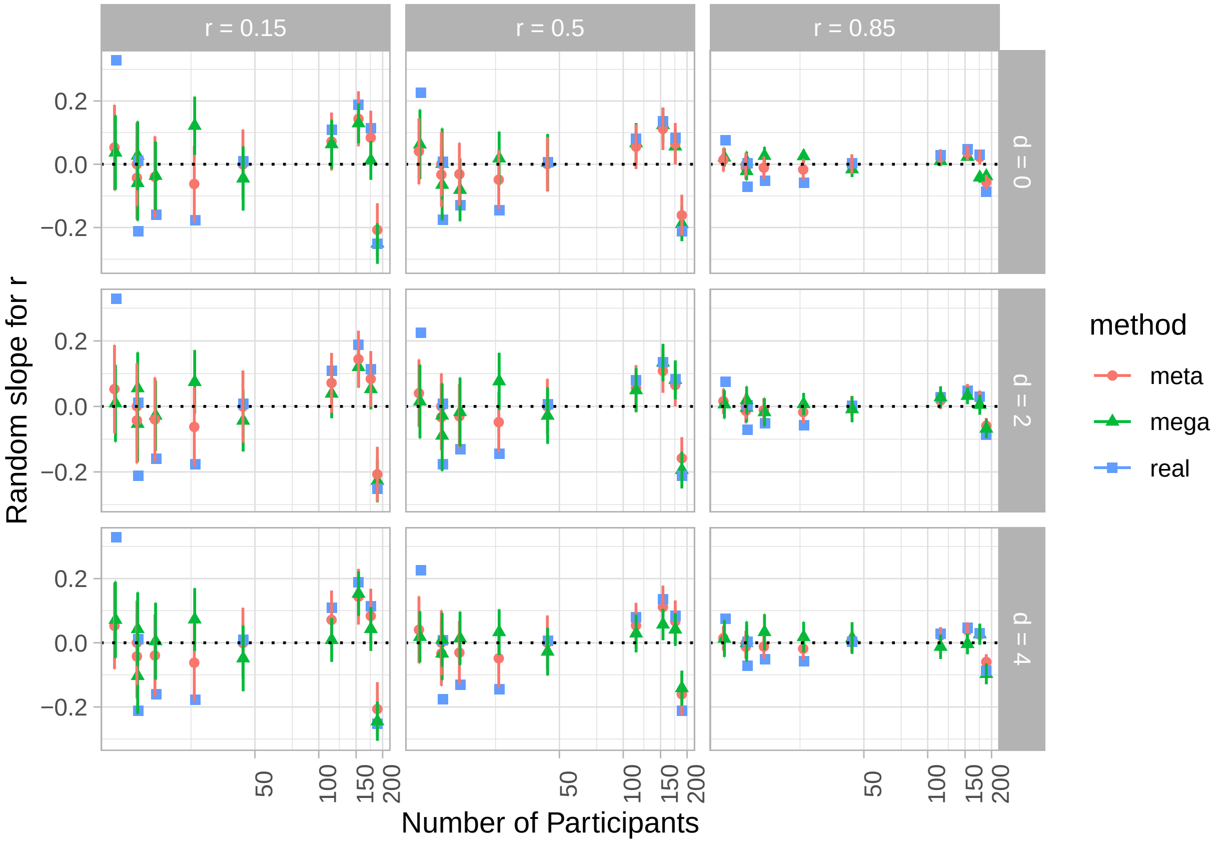

Figure 5: Random slopes per study for synthetic data as a function of participants' number in the analysis of the correlation between days of testing, according to two estimation methods: a meta-analysis (meta); a regression with the true individual-level data (mega). In each panel, we plot calculated d as a function of the true values of d and r in the data generation. Error bars represent standard errors for the fitted values. We can see how both mega- and meta-analysis improve their predictions as the number of participants increases.
